# Early transcriptional changes in the reef-building coral *Acropora aspera* in response to thermal and nutrient stress

**DOI:** 10.1186/1471-2164-15-1052

**Published:** 2014-12-02

**Authors:** Nedeljka Rosic, Paulina Kaniewska, Chon-Kit Kenneth Chan, Edmund Yew Siang Ling, David Edwards, Sophie Dove, Ove Hoegh-Guldberg

**Affiliations:** School of Biological Sciences, The University of Queensland, Brisbane, Qld 4072 Australia; Australian Institute of Marine Science, Townsville MC, Qld 4810 Australia; School of Agriculture and Food Sciences, The University of Queensland, Brisbane, Qld 4072 Australia; University of Queensland Centre for Clinical Research, The University of Queensland, Brisbane, Qld 4029 Australia; School of Plant Biology, University of Western Australia, Perth, WA Australia 6; Australian Centre for Plant Functional Genomics, The University of Queensland, Brisbane, Qld 4072 Australia; Global Change Institute and ARC Centre of Excellence for Coral Reef Studies, The University of Queensland, Brisbane, Qld 4072 Australia

## Abstract

**Background:**

Changes to the environment as a result of human activities can result in a range of impacts on reef building corals that include coral bleaching (reduced concentrations of algal symbionts), decreased coral growth and calcification, and increased incidence of diseases and mortality. Understanding how elevated temperatures and nutrient concentration affect early transcriptional changes in corals and their algal endosymbionts is critically important for evaluating the responses of coral reefs to global changes happening in the environment. Here, we investigated the expression of genes in colonies of the reef-building coral *Acropora aspera* exposed to short-term sub-lethal levels of thermal (+6°C) and nutrient stress (ammonium-enrichment: 20 μM).

**Results:**

The RNA-Seq data provided hundreds of differentially expressed genes (DEGs) corresponding to various stress regimes, with 115 up- and 78 down-regulated genes common to all stress regimes. A list of DEGs included up-regulated coral genes like cytochrome c oxidase and NADH-ubiquinone oxidoreductase and up-regulated photosynthetic genes of algal origin, whereas coral GFP-like fluorescent chromoprotein and sodium/potassium-transporting ATPase showed reduced transcript levels. Taxonomic analyses of the coral holobiont disclosed the dominant presence of transcripts from coral (~70%) and *Symbiodinium* (~10-12%), as well as ~15-20% of unknown sequences which lacked sequence identity to known genes. Gene ontology analyses revealed enriched pathways, which led to changes in the dynamics of protein networks affecting growth, cellular processes, and energy requirement.

**Conclusions:**

In corals with preserved symbiont physiological performance (based on *F*v/*F*m, photo-pigment and symbiont density), transcriptomic changes and DEGs provided important insight into early stages of the stress response in the coral holobiont. Although there were no signs of coral bleaching after exposure to short-term thermal and nutrient stress conditions, we managed to detect oxidative stress and apoptotic changes on a molecular level and provide a list of prospective stress biomarkers for both partners in symbiosis. Consequently, our findings are important for understanding and anticipating impacts of anthropogenic global climate change on coral reefs.

**Electronic supplementary material:**

The online version of this article (doi:10.1186/1471-2164-15-1052) contains supplementary material, which is available to authorized users.

## Background

Reef-building corals form obligate mutualistic symbiosis with unicellular photosynthetic dinoflagellates (genus *Symbiodinium*), which is based on the exchange of inorganic and photosynthetic compounds between the host and endosymbiotic algae [1–3]. Elevated sea surface temperatures (SSTs) have been recognized as the most important environmental variable leading to coral bleaching [4, 5], where SSTs of merely 1°C above the long-term summer maxima can result in mass bleaching events [4]. The future trajectory according to the Intergovernmental Panel on Climate Change (IPCC 2007), based on continuing increase in CO_2_ emission, unfortunately predicts a further increase in temperature during the 21^st^ century [6].

Another important anthropogenic stress factor is pollution of coastal waters. Reef corals can be negatively affected by nutrient enrichment such as dissolved Nitrogen (N) that may result in increased incidence of coral bleaching, leading to a further reduction in the abundance and distribution of reef-building corals [7–10]. In other cases, elevated amounts of N have been reported to positively impact coral growth and symbiont density [11, 12] and have been found to increase the percentage of algae released from host [13].

A number of studies have explored the effects of environmental stress on the physiological performance of the coral host, its associated microorganisms and the effect on changes in the gene expression levels [10, 14–22]. Novel genomic tools such as microarray analyses have brought an overview on the instant changes in the expression levels of hundreds, thousands or even more genes and at the same time, providing more information about the organism’s response to changes in the environment [14, 17, 23–26]. Next generation sequencing technologies provide a more comprehensive picture of changes in RNA expression profiles under different experimental conditions [27]. Only a few studies have applied the RNA-Seq approach to explore the effect of ocean acidification on coral calcification [22], the effect of heat and settlement inducers on the gene expression profiles of aposymbiotic larvae [28] and the molecular pathways involved in coral resilience to thermal stress [29]. Most recently, RNA-Seq revealed molecular pathways involved in heat-induced stress response of the coral *Porites astreoides* from thermally distinct reef habitats [30].

Here, we address the existing knowledge gap regarding the detection of early molecular changes after exposure to environmental stress at the level of the coral holobiont, which includes both coral and associated endosymbionts. On the global scale, thermal-driven stress and nutrient enrichment can negatively affect the well-being of corals, although the impacts may vary depending on stress level and coral species examined [8, 31, 32]. It is, therefore, important to describe the mechanisms and early warning signs at the molecular level before physiological changes leading to coral bleaching and potentially coral mortality are irreversible. In the present study, we exposed the reef-building coral *Acropora aspera* to 3-day thermal and nutrient (ammonium-enrichment) stress regimes and applied the RNA-Seq method (Illumina technology) to identify changes in the gene expression patterns between control and stress conditions. As part of the study, we identified genes/pathways in the coral holobiont involved in the response to short-term thermal and nutrient stress. Our results provide new insights into the transcriptional profiles of the coral holobiont and transcriptional regulation prior to bleaching. In addition, we identified a range of potential gene stress biomarkers that could be used for the detection of sub-lethal stress in reef-building corals.

## Results

### Physiological performance

**Figure 1:**
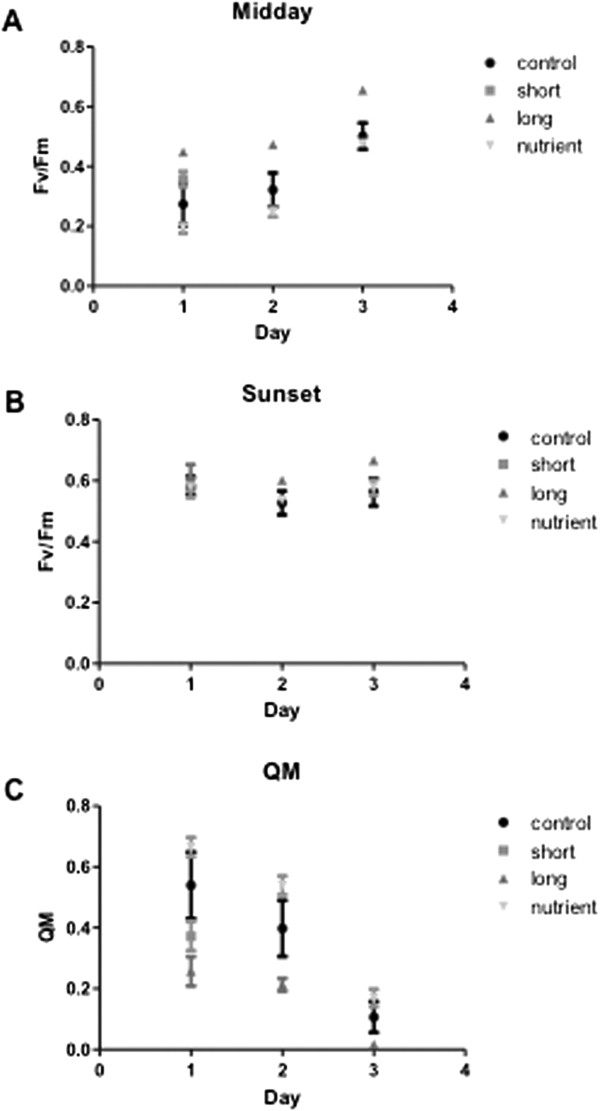
**Chl** ***a*** **fluorescence measurements.** Chl *a* fluorescence measurements of *A. aspera* at Heron island, southern Great Barrier Reef (23°27.625’S, 151°55.759’E) exposed to different ambient treatments; control (black circle), short term temperature stress (grey square), long term temperature stress (grey triangle) and nutrient stress (light grey triangle), **(A)** Maximum quantum yield (*F*v/*F*m) at Midday, **(B)** Maximum quantum yield (*F*v/*F*m) at Sunset and **(C)** Maximum pressure over photosystem II at noon (relative) (Qm). Error bars represent the standard error of the mean.

Photosynthetic efficiency of *Symbiodinium* in *A. aspera*, from pulse amplitude modulated fluorometry (PAM), revealed an overall increase in the maximum quantum yield (*F*v/*F*m) of photosystem II (PSII) at midday across all treatments (Figure 1A). Specifically, *F*v/*F*m at midday showed fluctuation as a consequence of both treatment (Kruskal-Wallis, H_2, 45_ = 20.57, *p* < 0.001) and also day (Kruskal-Wallis, H_2, 45_ = 13.67, *p* = 0.001), where the long thermal stress resulted in higher values of *F*v/*F*m compared to control (18% increase after 1-day, 46% after 2-day and 30% after 3-day period) and nutrient treatments (2.2-fold higher on day 1, 95% on day 2 and 34% on day 3). At sunset *F*v/*F*m varied with experimental treatment (Kruskal-Wallis, H_2, 45_ = 11.16, *p* = 0.005), with ~15% higher value in the long-term temperature stress treatment than in the control and nutrient treatments, and did not vary among days (Kruskal-Wallis, H_2, 45_ = 2.99, *p* = 0.224).

Maximum excitation pressure over PSII (Qm) varied with experimental treatment (Kruskal-Wallis, H_2, 45_ = 11.16, *p* = 0.038) and day (Kruskal-Wallis, H_2, 45_ = 22.73, *p* < 0.001), and showed overall decreased with time (Figure 1C). Corals exposed to nutrient treatment had a 2.6-fold higher Qm than the long-term temperature stress treatment on day 1, 35% and a 2.5-fold higher Qm than control and long term temperature stress on day 2 and a 2-fold higher Qm than the long term temperature stress treatment on day 3. Coral branches in the short-term temperature stress treatment did not change their photosynthetic activity compared to control branches at midday (*t*-test, t_1, 8_ = 5.08, *p* = 0.144) and sunset (*t*-test, t_1, 8_ = 1.28, *p* = 0.814) and there was no change in Qm (*t*-test, t_1, 8_ = 3014, *p* = 0.292) (Figure 1).

We monitored population density of *Symbiodinium* and the concentration of chlorophyll a under thermal and nutrient stress conditions as indicators of stress and coral bleaching. There was no difference in the algal cell density between treatment and control (Additional file 1A, *t*-test; *p* > 0.05). Also, chlorophyll a concentration (per surface area) did not differ between corals exposed to elevated temperatures and nutrient enrichment conditions (*p* > 0.05; Additional file 1B).

### Analyses of differentially expressed genes

**Figure 2:**
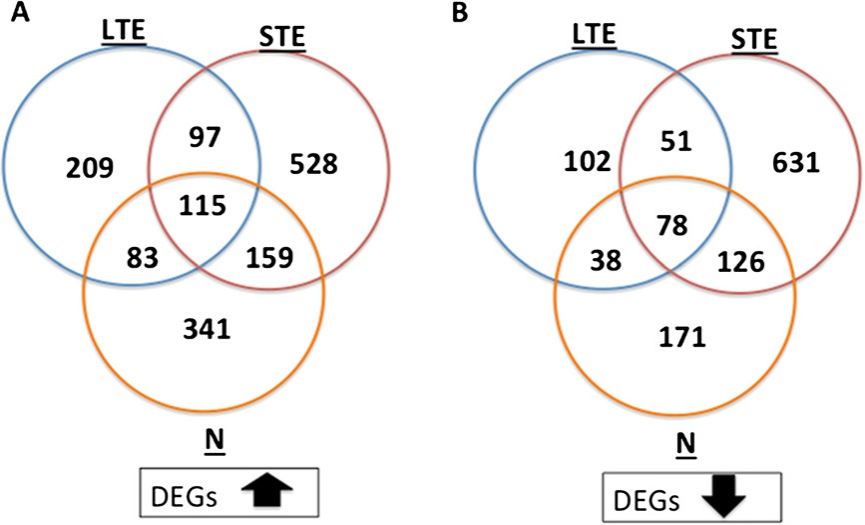
**DEGs with up and down-regulation.** Venn diagrams presenting the overlaps between DEGs with up- **(A)** and down-regulation **(B)** profiles in response to short (or 1-day) thermal stress (STE), long (or 3-day) thermal stress (LTE) and nutrient stress (N).

We compared gene expression profiles between control and treatment conditions that included thermal and nutrient stress using RNA-Seq technology. Illumina massively-parallel sequencing produced between 14–27 million reads (99 bp) per treatment. The analysis was performed using the Differential Kmer Analysis Pipeline (DiffKAP) method which progressively analyse the sequence data from *k*-mer to read and finally to gene level. The pipeline identified over a million differentially expressed reads (DERs) per treatment. Comparison of these DERs with the high quality annotated and non-redundant protein database, Swiss-Prot, resulted in successfully annotating around 10% of DERs (The DiffKAP run summaries containing all intermediate results are shown in Additional file 2: Table S1; Additional file 3: Table S2; Additional file 4: Table S3). After the standard DiffKAP analysis, two extra filtering steps on the results were applied for ensuring that other species in these complex metetranscriptome samples were not introducing false positive DEGs. The extra filtering was performed using the DiffKAP gene-centric summary file, which contains all annotated genes with their corresponding number of up- and down-regulated DERs in different columns. Annotated genes with fewer supporting DERs are possibly from other species within the coral holobiont, which are less abundant than coral and *Symbiodinium*. Using only annotated genes that contained 10 or more DERs as a cut-off value to obtain confident differentially expressed genes (DEGs) - corresponded to ~14.75-21.61% of all annotated genes - we identified: 3550 DEGs in short thermal stress exposure (STE) vs. control (CTRL); 1875 DEGs for long thermal stress exposure (LTE) vs. CTRL; and 1956 DEGs in Nutrient stress (N) vs. CTRL. Inconsistent expression profiles in particular genes may be due to various reasons, such as different expression levels of isoforms of a gene and less confident blast hits of short sequences. To prevent false positive DEGs we did additional filtering for DEGs with consistent expression profiles (containing all DERs annotated to the same DEGs with the same up- or down-regulation expression patterns under a specific experimental regime, i.e. either the up or down column in the DiffKAP gene-centric summary file will need to be zero). This reduced the list of DEGs by ~50%: 1785 DEGs in STE vs. CTRL; 773 DEGs for LTE vs. CTRL; and 1111 DEGs in N vs. CTRL. The overall ratios of up- and down-regulated DEGs between treatments are presented in Figure 2 and include the numbers of DEGs unique to specific treatments and also DEGs shared between treatments. A complete list of 115 up- and 78 down-regulated DEGs commonly expressed in all stress regimes is provided in Additional file 5: Table S4 and Additional file 6: Table S5, while some of the major DEGs shared between treatments are presented in Table 1. Overall, increased transcript abundance was noticed for some ribosomal proteins (40S 2, 3, 14 and 26; 60S 7 and 10), cytochrome c oxidase, cytochrome b, NADH-ubiquinone oxidoreductase, actin proteins, Fas apoptotic inhibitory molecule and programmed cell death protein of coral origin. Up-regulated algal DEGs included a number of photosynthetic genes of photosystem (PS) I and PSII, ATP synthase and cytochrome genes. Coral down-regulated DEGs contained different ribosomal proteins of 40S (small subunits 4, 17, 23, 24, 27, 28 and 29) and 60S (large subunits 13a, 14, 18a, 19, 24, 26, 27, 29, 31, 32, 34, 36, 37, 38 and 39), tubulin alpha and beta chains, GFP-like fluorescent chromoprotein and sodium/potassium-transporting ATPase subunit alpha of coral origin. Algal down-regulated DEGs included 60S ribosomal proteins (large subunits 13, 27, 37 and 39) and algal cytochrome c oxidase.

**Table 1:** **Up and down-regulated genes from RNA-Seq data**

Origin	Biological processes	Hit description (Acc. No.)	Regulation
Algal	Photosynthesis	Photosystem I P700 chlorophyll a apoprotein A1 (Q9XQV3)	UP
		Photosystem I P700 chlorophyll a apoprotein A2 (P58383)	
		Photosystem Q(B) protein (Q9TM69)	
		Photosystem II D2 protein (Q9TL00)	
		Photosystem II CP43 chlorophyll apoprotein (Q1XDD1)	
		Cytochrome b6-f complex subunit 4 (Q1XDE7)	
	Metabolism	Cytochrome c oxidase subunit 1 (Q02766)	DOWN
		ADP, ATP carrier protein (P25083)	
Coral	Respiratory chain, transport and electron transport	Cytochrome b (Q8SJB6)	UP
		NADH-ubiquinone oxidoreductase (Q35100)	
	Hydrogen peroxide catabolic process	Chorion peroxidase (P82600)	
	Ubiquitin conjugation pathway	E3 ubiquitin-protein ligase (Q9ULT8)	
	Metabolism	Cytochrome c oxidase subunit 1 (Q35101)	
	Transport	Sodium/potassium-transporting ATPase subunit alpha (P06687)	DOWN
		Electron transfer flavoprotein-ubiquinone oxidoreductase (Q5RDD3)	
		V-type proton ATPase 16 kDa proteolipid subunit (P63081)	
	Luminescence	GFP-like fluorescent chromoprotein (Q9U6Y6)	

Up- and down-regulated genes expressed in common across all stress regimes that might be explored further for potential use as stress biomarkers.

A number of DEGs were involved in the oxidative stress response and genes encoding antioxidant enzymes discovered in this study are listed in Table 2. The STE condition resulted in up-regulation of expression for superoxide dismutase, catalase, peroxidase, peroxiredoxin, glutathione S-transferase and thioredoxin, which are involved in the reduction of oxidative stress and capturing ROS. However, after the 3-day exposure to thermal stress (LTE), there was a down-regulation or lack of differential expression for the majority of antioxidant genes, whilst nutrient stress resulted mainly in the up-regulation of genes involved in suppressing the effects of oxidative stress.

**Table 2:** **Antioxidant DEGs up- and down-regulated**

Antioxidant enzymes	DEGs in STE	DEGs in LTE	DEGs in N
	(Gene Accession No.)	(Gene Accession No.)	(Gene Accession No.)
Superoxide dismutase	UP (O73872; Q8HXQ3; Q8HXQ4; P04178; Q8HXQ0)	0	UP (O73872; Q8HXQ3; Q8HXQ4; O46412; Q0IIW3; P80174; P80566)
Catalase	UP (O62839; Q9PWF7; P17336; Q9PT92; Q27487; Q9XZD5; P04040; O93662; Q64405; O77229; Q2I6W4; P00432; P90682)	0	UP (Q9PWF7; O62839; Q4AEI3; P22079; Q98234)
Peroxidase	UP (P82600; Q9VEG6; P05164; P22079)	UP (Q9VEG6; P82600)	UP (P82600; Q4AEI0; Q9VEG6)
		DOWN (Q4AEH2)	
Peroxiredoxin	UP (Q8T6C4; Q63716; Q9GLW9; Q9BGI1)	DOWN(Q9V3P0; P34227; O35244; Q9Z0V5)	UP (Q9Z0V5)
			DOWN (Q90384; Q9V3P0; P34227; Q6DV14)
Glutathione S-transferase	UP (Q9N1F5; Q3T100)	0	UP (Q3T100)
Thioredoxin	UP (P20108; Q99MD6; Q5NVA2; Q9NNW7)	DOWN (P83877)	DOWN (Q99MD6; O89049; P91938; B9A1H3; Q86VQ6)

A list of antioxidant DEGs up- and down-regulated by STE, LTE and N treatments including their Gene accession Numbers in the reef building coral *A. aspera*. Only DEGs containing 10 or more DERs are included.

### Validation of the DiffKAP method by quantitative PCR

We validated the DiffKAP method by real-time quantitative PCR (qPCR) and compared transcript abundance of nine randomly selected DEGs, characterized by different levels of expression (based on Ratio of Medians for Treatment vs. CTRL, Additional file 7). GeneBank Accession Numbers of the targeted genes are provided in Table 1. Changes in expression of the nine coral and algal genes after a 3-day period of heat and nutrient stress were monitored by qPCR and presented in Additional file 7. Results revealed the positive correlation in gene expression changes (up- or down-regulation) identified by both methods, qPCR and RNA-Seq, and verified the appropriateness of the *k*-mer approach as implemented in the DiffKAP method.

**Figure 3:**
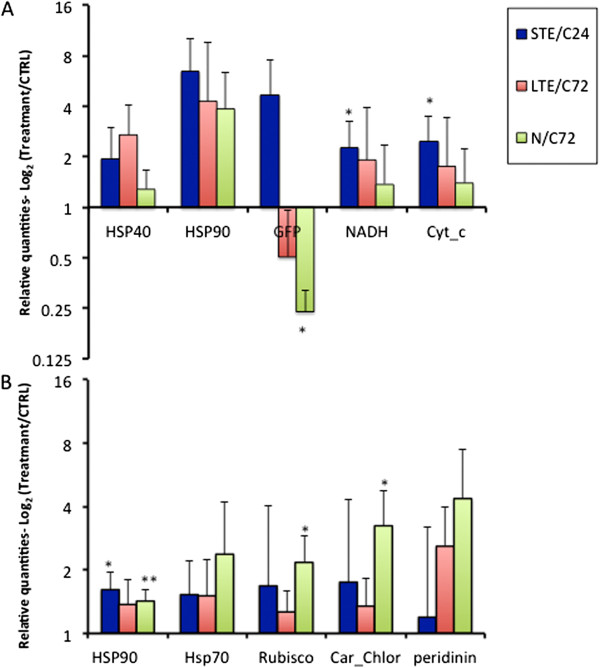
**Relative gene expression.** The relative expression of targeted genes in the STE, LTE and N regimens for selected coral **(A)** and dinoflagellate **(B)** genes by qPCR. Relative quantities were normalized to two most stable reference genes with pairwise variations (*V*) for minimum of 2 or 3 reference genes (*V*2/3) below 0.15. The mean expression level was obtained as ratio of treatment vs. control (C24 correspond to control at 24 h and C72 - control at 72 h) for five biological replicates. The statistical difference between means is indicated as *P < 0.05 or **P < 0.01.

In order to explore potential stress biomarkers, we also evaluated the level of transcript abundance in several candidates proposed by the DiffKAP analyses (Table 3) and from previous studies [15]. Coral and dinoflagellate molecular chaperones, *Heat Shock Proteins* (HSPs), showed increased levels of expression, but significant change was observed only for algal *Hsp*90 in STE and N, but not LTE (Figure 3). Up-regulation of *Symbiodinium Hsp*90 was significant after 1 day of exposure to elevated temperatures (1.6-fold; *t*-test: t_4_ = -3.98, *p* = 0.016) and ammonium enrichment conditions (1.4-fold; *t*-test: t_4_ *= -*4.90, *p* = 0.008). Significant down-regulation was observed for coral *green fluorescent protein (GFP)-like fluorescent chromoprotein* under nutrient stress conditions (0.24-fold; t_4_ = -3.15 *p =* 0.034). During 1-day thermal stress increased expression was noted for algal *cytochrome c oxidase subunit 1* (2.4-fold; *t*-test: t_4_ = -3.15 *p =* 0.034) and *NADH-ubiquinone oxidoreductase* (2.2-fold; *t*-test: t_4_ = -2.91 *p =* 0.034). The changes in targeted algal genes were significant under nutrient enriched conditions showing up-regulation for *ribulose bisphosphate carboxylase* for a 2.1-fold increase (*t*-test: t_4_ = -3.50, *p =* 0.024) *and caroteno-chlorophyll a-c-binding protein* for a 3.2-fold rise (*t*-test: t_4_ = -3.34, *p =* 0.028).

**Table 3:** **qPCR analyses of DEGs**

Symbol (C-Coral or A-algae)	GenBank accession number	Annotation	Species with the best BLAST (GenBanAcc)	E-value
*Hsp90 (C)*	JT002485	Heat shock protein HSP 90-alpha	*Rattus norvegicus* (P82995)	4.00E-11
*GFP (C)*	EZ013774	GFP-like fluorescent chromoprotein	*Anemonia majano* (Q9U6Y6)	3.00E-09
*NADH (C)*	JT002895	NADH-ubiquinone oxidoreductase	*Metridium senile*(O47498)	2.00E-10
*Tyr (C)*	JT013311	Tyrosyl-tRNA synthetase	*Danio rerio* (Q6TGS6)	2.00E-09
*Cyt_c (C)*	JT015689	Cytochrome c oxidase subunit 1	*Metridium senile* (Q35101)	2.00E-11
*Rubisco (A)*	GAFO01026698	Ribulose bisphosphate carboxylase	*Symbiodinium* sp. (Q41406)	6.00E-13
*Peridinin (A)*	JN602625	Peridinin-chlorophyll a-binding protein	*Symbiodinium* sp. (P51874)	6.00E-11
*Car_Chl (A)*	FN646420	Caroteno-chlorophyll a-c-binding protein	*Amphidinium carterae* (P55738)	2.00E-08

GenBank accession number, the best BLASTx (against Swiss-Prot database) and functions of selected highly abundant DEGs based on *k*-mer analyses used for qPCR analyses.

### Functional profile and gene ontology (GO) enrichment analyses

In coral samples exposed to thermal and nutrient stress conditions a number of significantly enriched molecular pathways were detected (Table 4). Examples of enriched pathways after 1-day thermal stress included: *ribosome*, *proteasome*, *ubiquitin-mediated proteolysis*, *calcium signalling pathway*, *citrate cycle* also known as *TCA* (tricarboxylic acid) or Krebs cycle; whilst thermal stress lasting for a 3-day period resulted in the enrichment of the following pathways (Table 4): *ribosome*, *valine, leucine and isoleucine degradation*, *insulin signalling pathway*, *ubiquitin mediated proteolysis* and *spliceosome*. Nutrient stress resulted in the enrichment of ten pathways, with two pathways, *ribosome* and *ubiquitin mediated proteolysis,* commonly enhanced by all stress regimens.

**Table 4:** **Gene enrichment analyses**

Significant pathways	Condition	No of genes	No of KEGG genes in pathway	Fold enrichment	Corrected ***P***-value
***Ribosome***	**STE**	41	193	3.03	6.90E-09
***Proteasome***		17	50	3.51	2.30E-03
***Ubiquitin mediated proteolysis***		32	143	2.26	3.50E-03
***Calcium signaling pathway***		35	193	2.14	4.70E-03
***Citrate cycle (TCA cycle)***		16	31	3.17	5.60E-03
***Ribosome***	**LTE**	46	193	2.15	1.71E-05
***Valine, leucine and isoleucine degradation***		25	53	2.37	4.28E-03
***Insulin signaling pathway***		40	142	1.87	1.14E-02
***Ubiquitin mediated proteolysis***		37	143	1.90	1.35E-02
***Spliceosome***		36	136	1.87	2.02E-02
***Ribosome***	**N**	47	193	2.61	8.80E-09
***Citrate cycle (TCA cycle)***		19	31	2.91	5.10E-03
***Ubiquitin mediated proteolysis***		37	143	2.08	3.50E-03
***Valine, leucine and isoleucine degradation***		23	53	2.45	3.50E-03
***Focal adhesion***		40	204	1.92	1.20E-02
***Endocytosis***		39	236	1.89	1.10E-02
***Spliceosome***		33	136	2.01	1.70E-02
***Lysosome***		26	127	2.12	1.70E-02
***Insulin signaling pathway***		34	142	1.89	4.20E-02
***Oocyte meiosis***		25	114	2.13	4.40E-02

Gene enrichment analyses for coral samples exposed to thermal and nutrient stress. Significant pathways involved in short (STE), long-thermal (LTE) and nutrient (N) stress response had corrected *P*-value by Benjamini *P* < 0.05.

**Figure 4:**
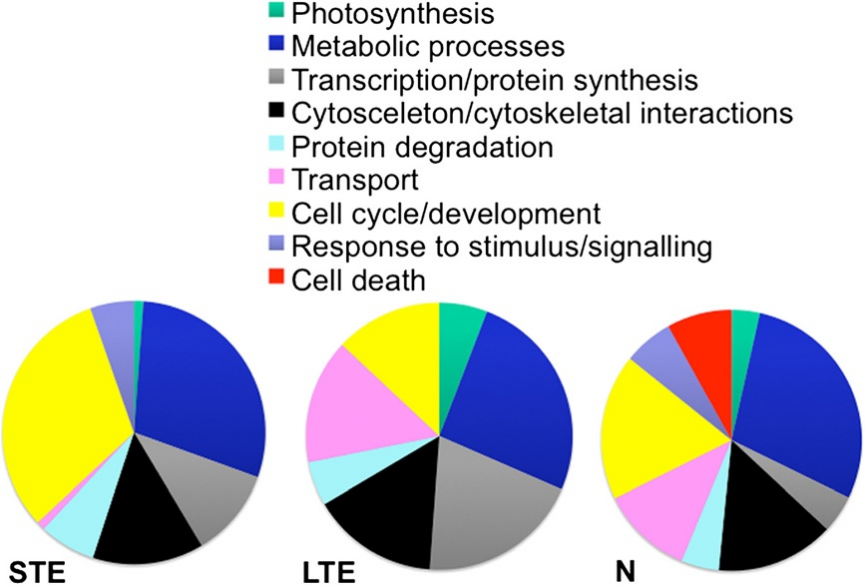
**Biological processes enriched by stress.** Biological processes enriched in the coral host *A. aspera* exposed to heat and nutrient stress.

Examples of enriched GO categories after 1-day thermal stress conditions (corrected *P*-value by Benjamini *P* < 0.001) included 27 biological process (BP) categories, 13 molecular function (MF) and 9 cellular component (CC) categories. The 3-day thermal stress caused the enrichment of 16 BP categories, 7 MF and 8 CC categories. After exposure to a nutrient stress regime, we observed enrichment in 46 BP categories, 18 MF and 19 CC categories. BP-enriched categories included: photosynthesis, metabolic processes, cell cycle and development, transport (after 3-day stress), protein degradation, and cell death (under nutrient stress) as presented in Figure 4. Top 10 enriched BP, MF and CC categories based on the fold enrichment are presented in Additional file 8: Table S6; Additional file 9: Table S7; Additional file 10: Table S8.

### Taxonomic analyses

**Figure 5:**
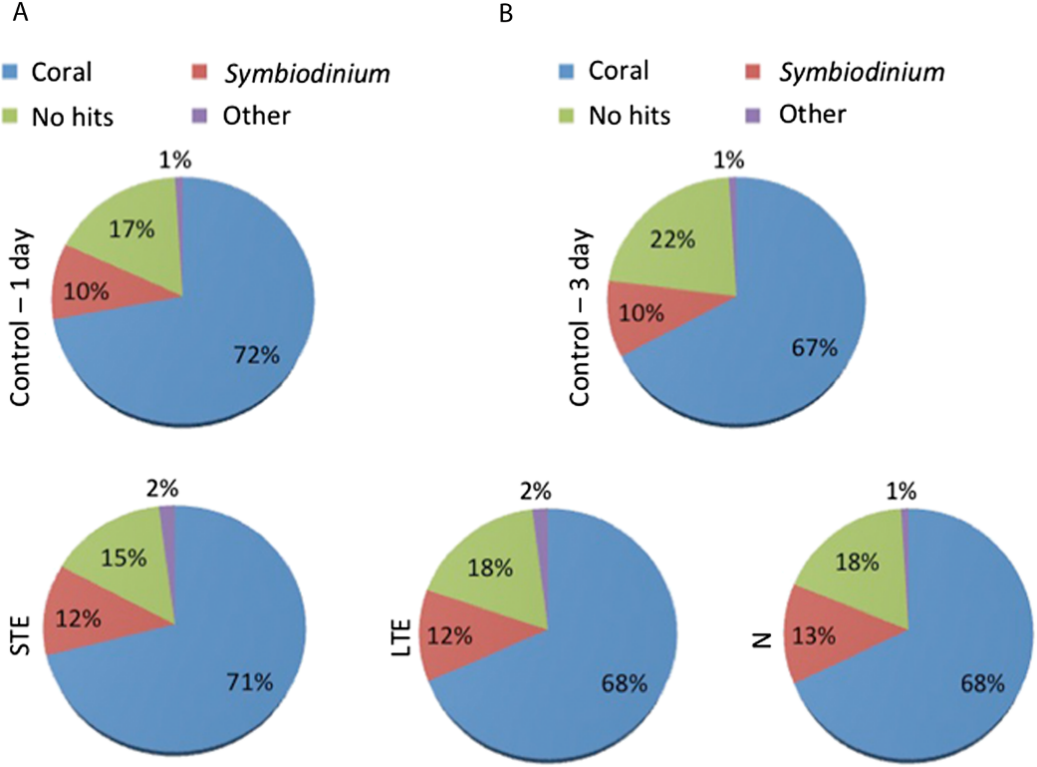
**Taxonomic composition.** Taxonomic composition within the coral holobiont obtained by aligning the millions of short reads to publicly available sequence databases, after 24-h **(A)** and 72-h **(B)** period in treatments (1-day and 3-day thermal stress and nutrient stress) and control (CTRL 24 and CTRL 72).

Taxonomic composition of the coral holobiont samples was obtained after aligning millions of short sequence reads to publicly available sequence databases and the *Symbiodinium de novo* transcriptome assemblies generated by our group [33]. The total number and percentage of reads aligning to the various databases used in this study are provided in the Additional file 11: Table S9 and Additional file 12: Table S10. The taxonomic analyses revealed dominant presence of coral transcripts (up to 72% of sequences) under both control and stress conditions (Figure 5). The second most dominant group with attribution, comprising ~10-12% of transcripts, corresponded to the *Symbiodinium* databases and 1-2% of transcripts had hits to sequences belonging to environmental samples and various organisms such as bacteria, viruses, fungi, plants and algae, invertebrates, and even humans. The remaining 15% to 22% of short reads lacked a significant match in the databases (Figure 5). Exposure to stress resulted in overall increased abundance of bacterial and viral transcripts up to ~10-fold compared to control conditions, despite our sequencing being limited to poly-A-containing mRNA molecules (Additional file 11: Table S9 and Additional file 12: Table S10).

## Discussion

Molecular and physiological responses represent the first line of defense in reducing potentially harmful effects of unfavorable conditions in the environment. Our understanding of the underlying mechanisms of the coral holobiont stress response, however, is still limited. Gene expression studies offer a powerful approach for better understanding the complexity of how organisms respond to changes in their environment. Coral communities are facing increasing thermal stress, with mass coral bleaching often triggered by relatively small temperature increases over long-term summer maxima [4]. On the Great Barrier Reef, natural bleaching conditions were described after a 5-day period of temperatures between 31°C and 32°C [5, 34], although coral susceptibility to thermal induced bleaching may vary among reefs with different historical thermal backgrounds [35–38]. Studies have shown that both coral host and *Symbiodinium* types play important roles in coral susceptibility to bleaching [39–43]. In the present study, we applied the sub-lethal level of thermal stress of 30°C (+6°C) that was just below the bleaching threshold, as well as ammonium enriched conditions. These conditions allowed corals to maintain healthy photosynthetic fitness based on elevated dark-adapted *F*v/*F*m, pigment concentration and symbiont cell density (Figure 1 and Additional file 1). As we terminated the heating experiment after 1 and 3-day periods, we cannot know with the certainty that the reduction of *F*v/*F*m or decrease in the number of dinoflagellates as indication of coral bleaching would occur with prolonged exposure to heat. However, it has been shown that heat stress for a 3–4 day period often results in symbiont cell density reductions [44, 45]. Similarly to our results, Fisher et al. reported lack of significant changes in the PSII activity for the reef-building coral *A. aspera* from GBR after 3 days of heat stress, followed by a drop in the PSII activity after a 4-day period of prolonged heat stress [40].

RNA-Seq has been shown to be a reliable tool for the quantification of the gene expression levels and comparable to qPCR levels [46], whilst in this challenging metatransciptome environment containing coral, dinoflagellates and other associated organisms the identification of DEGs was a difficult task. We applied a novel approach for analyzing metatranscriptomes called DiffKAP. This innovative bioinformatics method uses a pairwise comparison between Illumina read *k*-mers from control and treatment samples to estimate the changes in the level of gene expression. From DEGs revealed here, hundreds of up- and down-regulated genes of the coral holobiont have been recognized for potential use as stress biomarkers (Figure 2). These genes, differentially expressed during short-term exposure to +6°C-elevated temperatures and ammonium-enriched conditions, are involved in biological processes related to photosynthesis, respiration, transport and protein degradation, including genes from PSI and PSII (Table 1). During coral exposure to elevated temperatures, the photosynthetic machinery of endosymbotic dinoflagellates is susceptible to heat impairment [45, 47], especially the D1 protein [48, 49], resulting in a decline in PSII functionality [50, 51]. Under sub-lethal levels of heat and nutrient stress, we induced transcription of a number of photosynthetic genes from PSI and PSII including expression of PSII D2 protein, confirming their sensitivity to heat-induced changes and role in the stress response (Table 1). Photosynthetic genes have been shown to play a role in minimizing photoinhibition of PSII and scavenging ROS during light-induced stress in plants [52]. In *Symbiodinium* cultures an initial increase in expression of photosynthetic genes (*psb*A and *psa*A) during exposure to sub-lethal heat stress of 29°C and 31°C was followed by decline after reaching the upper-thermal threshold at 32°C [53]. However, transcripts of organelle genes like photosynthetic genes of the dinoflagellate *Lingulodinium*, often have a polyuridylylated 3′ residue [54], which may affect the accuracy of gene expression profiles when targeting poly-A-containing mRNA molecules using standard RNA extraction kits. The presence of a polyA tail in the transcripts of some chloroplast-encoded genes is found to be a signal of RNA degradation [55] and therefore, increase in their expression may suggest transcript degradation and destabilization [56].

We detected a trend in up-regulation of coral genes *Hsp*90 and *Hsp*40, although without significant changes (Figure 3; *t*-test, *p >* 0.05) and an ~2-fold increase in abundance of algal HSP transcripts, which is similar to changes in *Symbiodinium* gene expression reported by others [15, 21, 57–60]. Heat shock proteins (HSPs) are molecular chaperones sensitive to temperature changes and in the coral-algal symbiosis their expression may vary depending on time and the stress regimes [15, 21, 61, 62]. Previous studies have reported up-regulation of coral HSPs transcripts after short-term heat stress [17, 28], but also a lack of change in expression levels of HSPs [62, 63], especially after prolonged stress [28]. In other studies, down-regulation of algal *Hsp*90 expression was observed after more gradual increase in temperatures (~0.1°C per hour) during a 5-day period [15, 21]. Here, initial up-regulation of algal *Hsp*90 after 1-day and lack of significant changes after a 3-day period may actually suggest a way of acclimatization to prolonged heat stress. Also in other dinoflagellates, a majority of analyzed genes showed an even transcript abundance, with a small number of genes having increased expression levels, suggesting that gene regulation was occurring at the translational or post-translational levels [64].

Down-regulation of green fluorescent protein (GFP)-like homologues was observed after the 3-day thermal and nutrient stress regimes (Figure 3). The decrease in expression of GFP-like homologues under thermal stress has been previously reported [17, 65–67]. Similarly, Roth and Dehain (2013) confirmed a decrease in GFP concentration and fluorescence in the branching coral *Acropora yongei*, as an indicator of coral health decline before any bleaching signs could be observed [68]. Consequently, these GFP proteins should be further explored for their potential use in the development of bioluminescence chips for the evaluation of coral health.

Gene pathway enrichment analyses revealed a number of pathways involved in the coral holobiont response to changes in thermal and nutrient environmental conditions. *Ribosome*, *ubiquitin-mediated proteolysis* and *valine, leucine and isoleucine degradation* pathways, associated with the processes of protein synthesis and protein degradation were enriched during both thermal and nutrient stress regimes. This indicates protein perturbations and *de novo* protein synthesis, and overall changes in protein networks affecting the processes related to the cell cycle, immune responses, signal transduction, development and differentiation [69–72]. Protein degradation via the *ubiquitin/proteasome* pathway targets many short-lived regulatory proteins, such as cell cycle regulatory proteins, allowing the quick transitions between cell cycle stages [73]. We also observed a number of differentially expressed ribosomal genes of coral and microbial origin indicating shifts in the ribosomal activities (Additional file 9: Table S7 and Additional file 10: Table S8). In coral larval transcriptomes, decreased expression of ribosomal genes was observed after a 2-day [28, 74] and a 5-day exposure to heat stress [28]. Our finding of *calcium-signaling* pathway enrichment in STE is supported by a previous study, in which intracellular calcium accumulation was induced by heat [75]. The importance of calcium signaling for dinoflagellate-cnidarian symbiosis has been recently proposed with the discovery of conserved calcium-dependent protein kinase genes in various symbiotic dinoflagellates [33].

After a 3-day period of both thermal and nutrient stress, our analyses detected enrichment of the *spliceosome* pathway, which demonstrates the activation of transcriptional machinery and the processes of splicing. Energy demand was also increased, which involved pathways such as the *tricarboxylic acid (TCA) cycle*, an essential aerobic pathway important in an organism’s defence mechanism, as well as reactive oxygen species (ROS) detoxification and metabolism [76]. Others have also reported up-regulation in gene expression of TCA cycle enzymes by heat stress [21]. *Insulin signaling* pathways were also enriched, suggesting the role of metabolic processes such as glucose and lipid metabolism in the stress response. The insulin protein family is evolutionarily ancient [77] and insulin-related proteins have been found playing a role in signaling and growth in cnidarians [78].

During nutrient stress exposure, our results revealed the enrichment of the pathway related to *ocyte meosis* and the *endocytosis* pathway that is involved in molecule uptake. *Oocyte meiosis* pathway enrichment indicates gamete production was stimulated in corals. Oocyte formation was also increased by nutrient enrichment in the brooding coral *Stylophora pistillata*, although the final number of live planulae was reduced [79]. Ourresults show that catabolic processes are boosted by nutrient stress, as are ion transport and enzyme activities (Additional file 8: Table S6). Ammonium enrichment at the same concentration as was used in this study (20 μM) resulted in increased mortality of coral larvae [80]. Here, the impact of a short period (3 days) of elevated ammonium concentrations (10-20× higher than in a typical healthy coral reef environment) was not as detrimental, although apoptotic biological processes were enriched by nutrient stress, suggesting that the tipping point leading to apoptosis had been reached (Figure 4). This is possibly due to ammonium uptake by the host and largely by symbiotic dinoflagellates [19] and also natural variation in N concentration, as corals are exposed to pulses of elevated amounts of N released by residential fishes [11]. The up-regulation in expression of the algal genes *ribulose bisphosphate carboxylase* and *caroteno-chlorophyll a-c-binding protein* under nutrient enriched conditions (Figure 3) suggests the strengthening photosynthetic capacity of coral symbionts. Similarly, feeding of the reef-building corals had a positive effect on symbiont photosynthetic capacity during thermal stress, due to boosted food provision from the coral host to algal symbionts [81].

Although physiological performance was stable (Figure 1), the signs of oxidative stress were developing at the molecular level as RNA-Seq data showed enrichment of BP related to oxidative stress and cell death (Figure 4). Dysfunction of the coral-algal symbiosis, leading to a breakdown of the symbiotic partnership often starts with the production of ROS and oxidative stress [82]. In corals, it has been proposed that antioxidants scavenge ROS produced during heat stress, improve photosynthetic activity and decrease bleaching events [83]. Interestingly, higher numbers of antioxidative genes have been discovered in *Symbiodinium* compared to related, but non-symbiotic organisms [33, 84], indicating the enhanced capacity to endure oxidative stress. Our results show an up-regulation in expression of antioxidant genes under STE and nutrient stress conditions (Table 2), indicating oxidative stress response, in line with the findings of DeSalvo *et al.* [85]. Several forms of superoxide dismutase and catalase were enriched, which is consistent with their role as the first line of defense against oxidative stress and the protection of coral-algal symbiosis from ROS [86, 87]. During the LTE treatment, however, expression of superoxide dismutase and catalase was not affected, whilst peroxiredoxin and thioredoxin genes were down-regulated, suggesting possible acclimatization to elevated temperature by prolonged heat stress. Barshis *et al.* [29] also noticed reduced expression of antioxidant Cu-Zn superoxide dismutase in thermo-tolerant corals compared to thermo-sensitive types. In addition, our results show that after a 3-day exposure to thermal stress, corals in fact increased *F*v/*F*m and consequently their photosynthetic capacity (Figure 1), further pointing to possible photo-acclimation.

Multicellular organisms like corals can be regarded as metaorganisms comprised of the host and associated prokaryotic and eukaryotic organisms [88]. In the case of the coral holobiont, this entity is presented as the partnership between corals, symbiotic dinoflagellates and other organisms including microbiota, viruses and fungi [89]. Our taxonomic analyses of the reef-building coral *A. aspera* revealed dominance of transcripts with coral (~70%) and *Symbiodinium* origin (~10-13%) including sequences of unknown origin (~15-20% of total reads) with no significant matches to the sequence databases (Figure 5). This is possibly due to a large portion of sequences lacking hits to known genes and also the short sequencing reads produced by next-generation sequencing [90, 91]. Here, additionally, we identified the presence of 1-2% of transcripts from other organisms including bacteria, archaea, fungi, viruses and even human. These results can be explained by database bias due to a high proportion of characterized sequences from some model organisms, possible gene orthologs and also the existence of poly A bacterial and viral transcripts.

Exposure to stress has been demonstrated to lead to changes in the microbiota composition and susceptibility to coral diseases [20, 92], where heat stress can lead to shifts in microbial communities between healthy and bleached corals [93]. Here, stress resulted in an overall increased abundance of poly A bacterial and viral transcripts up to ~10-fold compared to control conditions. A recent proteomics study revealed a 100-fold increase in a viral replication protein in the coral *Stylophora pistillata* after heat stress treatment [94]. It is difficult, however, to distinguish the exact changes in the transcript abundance of specific groups of organisms due to the high proportion of unknown sequences. This will remain a recurring issue until sequencing projects, which will cover all species existing in the coral holobiont are completed.

## Conclusions

Elevated sea temperatures, ocean acidification and eutrophication are recognized as major factors in destabilizing the cnidarian–dinoflagellates symbiosis and consequently coral reef ecosystems globally [4, 95]. This novel study uses Illumina RNA-Seq technology to investigate the coral holobiont transcriptomic response to short-term heat and nutrient stress. We provide the comprehensive coral transcriptomic profile of the early changes happening at the molecular level before physiological and phenotypic changes can be observed. These results provide insights into the biological processes and pathways enriched by heat and ammonium augmentation such as *ribosome* and *ubiquitin-mediated proteolysis* pathways, resulting in a range of modifications in the protein networks. Our transciptomics data include the first pool of potential stress biomarkers comprised of coral and algal genes for detecting the early signs of stress in the coral holobiont. In the future, additional studies should apply “clinical trial” procedures, using different coral species and stress regimes to evaluate the biomarkers’ sensitivity and potential for technological application, including the development of environmental monitoring tools such as “molecular stress kits” for corals. To conclude, this study provides new insights critical for future prediction and protection of coral reefs from global climate change.

## Methods

### Experimental design

Coral fragments (7 cm long) of *A. aspera* (five colonies) harbouring *Symbiodinium* C3 genotype [96] were collected in the winter from the reef flat at Heron Island, Great Barrier Reef, Australia (23°25′S; 152°07′E) during the low tide (June 2010). After collection, the coral fragments were immediately transferred to flow through aquaria, fixed to racks and then returned to the reef flat to acclimatise for 2 weeks. Experimental treatments were carried out in 15 L tanks supplied with unfiltered sea water from the reef flat with a flow rate of 0.4 L per min. Tanks were independently heated and water pumps (250 L per hour) were used within each tank, to provide additional flow and support even heating. The experiment started at noon and included: [1] short thermal stress exposure (STE) reaching 6-7°C above ambient temperature after 6 h (~1°C increase per h, 30 ± 1°C) and lasting for a 1-day period; [2] long thermal stress exposure (LTE) reaching 6°C above ambient temperature after 12 h (~0.5°C increase per h, 30 ± 1°C) and lasting for a 3-day period and finally a control group stable at 23–24 ± 1°C; ambient temperature over a 24-h period (C24) and a 72 h-period (C72). Coral branches from five different colonies (five biological replicates) were randomly distributed across flow through aquaria (three aquaria per treatment). In the case of nutrient stress condition, five coral branches (n = 5) were exposed to elevated ammonium concentration in a 15-L tank with recirculating system, where spiking with 1 M NH_4_Cl (once a day) reached 20 μM after 3 days.

Two warming events reported on the Great Barrier Reef (GBR) that resulted in coral bleaching included temperatures between 31-32°C over a 5-day period and between 25°C and 32°C over a 24-h period [5, 34, 97]. Here, we aimed to avoid coral bleaching and therefore we applied sub-lethal thermal stress condition with maximum of 30°C (that was 1-2°C lower than during bleaching events) for 1-day and 3-day periods. Temperatures above 30°C led to photosynthetic dysfunction in *Symbiodinium* cultures [98]. In addition, the long-thermal threshold during 1980s for the reef flat at Heron Island has been reported to be ~29.5-30°C [99]. This concentration (ammonium-enrichment: 20 μM) represents the nutrient stress condition corresponding to 10–20 times greater ammonium concentration than found in nature [8, 100]. For each replicate aquarium water temperature was measured every 2 min using StowAway TidbiT Loggers (Onset Computer Corporation, USA). The maximal temperature applied here was 30°C, which is approximately 6-7°C higher than mean seawater temperature during the course of the experiment.

Additional details regarding experimental set up are provided in Additional file 13.

### Chl a fluorescence and maximum excitation pressure over photosystem II (Qm)

To measure the photosynthetic activity of *Symbiodinium* in *A. aspera* exposed to the different treatments Chl *a* fluorescence measurements were taken using a submersible fluorometer (Diving-Pam, Walz, Effeltrich, Germany). Measurements of the maximum quantum yield (*F*v/*F*m) were taken at midday (12.00) and at sunset (17.30) for days 1, 2 and 3. Upper parts of *A. aspera* branches in experimental aquaria were selected for measurements. Maximum excitation pressure over photosystem II (Qm) was defined as: Qm =1 – [(*ΔFv/Fm’* at midday)/(*Fv/Fm* at sunset)] following methods of Iglesias-Prieto et al. [101].

### *Symbiodinum*cell density and pigment composition

The algal cell density within the tissue of *A. aspera* samples was done by airbrushing frozen coral fragments in 14 ml of 0.06 M phosphate buffer (pH 6.65); followed by centrifugation of the homogenate at 4,000 × *g* for 5 min, re-suspension of pellet in filtered sea-water (0.45 μm) and separation in aliquots. An aliquot was used for the estimation of *Symbiodinium* cell density using 12 randomly picked replicates in the cell counter (Sedgewick-Rafter cell). The cell counts were normalized to the surface area (cm^2^) of each coral branch obtained using the melted paraffin method [13]. For pigment extraction, additional centrifugation (4000 × *g* for 5 min) was applied on the aliquot used for the pigment analyses followed by the re-suspension of pellet in 1 ml of 100% cold methanol, followed by 10 min sonication on ice cold water and centrifugation (4000 × *g* for 5 min). Supernatant was transferred to new tube and kept on ice. Additional 1 ml of 100% cold methanol was added to the pellet and the pigment extraction step was repeated until all pigment extract was removed from pellet (usually after 3–5 subsequent steps). The filtered pigment extraction (0.45 μm) was used for pigment separation in a Shimadzu SCL – 10 HPLC linked to a Shimadzu SPD – M10A photodiode array detector, using the column and method described by Zapata et al. (2000) with solutions A (methanol: acetonitrile: ammonium acetate, 50:25:25 v:v:v) and B (methanol: acetonitrile: acetone, 20:60:20 v:v:v). Pigments concentrations were obtained using the appropriate standards (chlorophyll a) and normalized to the surface area (cm^2^).

### Total RNA extraction and sequencing

Total RNA was extracted from coral branches using a small fragment (0.5-1 cm long) of coral nubbins that were cut with a bone cutter and ground in liquid nitrogen. The obtained powder was put in Trizol, homogenised with a hand homogenizer (Tissue-Tearor, Biospec products, Inc.) and centrifuged for 3 min at 13,000 × *g* at 4°C. The aqueous phase was then used for the extraction of total RNA with RNeasy kit (Invitrogen, Australia) following the manufacturer’s instructions. The RNA quantity and integrity was analyzed using a NanoDrop ND-1000 spectrometer and an Agilent 2100 Bioanalyzer (RIN > 7). Equal concentrations of a high quality RNA from biological replicates (n = 5) were pooled together and used for sequencing using the Illumina GA II 7Sequencing System (using the Illumina® TruSeq® RNA Sample Preparation Kit) by Australian Genome Research Facility Ltd (AGRF) that resulted in over 6 million reads per replicate (four technical replicates) per sample.

### Bioinformatics analyses - analyses of differentially expressed genes

Standard RNA-Seq analysis relies on mapping individual short sequence reads to a reference genome or transcriptome and then applying statistical tests to identify DEGs. However, as there is no reference transcriptome of *A. aspera* nor for the entire coral holobiont, it is challenging to apply the standard RNA-Seq analysis methods such as edgeR [102], DEGseq [103] or Cuffdiff [104]. *De novo* assembly of the RNA-Seq data requires large computing resources with high data coverage and is notoriously sensitive to sequencing error and chimeric assembled contigs [105], especially for metatranscriptome data. Therefore, we applied a DiffKAP method that allowed us to identify DEGs between two samples without using a reference.

The DiffKAP method is based on the fact that *k*-mers are the building blocks of reads which form a gene. By reducing each read to component *k*-mers and comparing the relative abundance of these sub-sequences, it overcomes statistical limitations of whole read comparative analysis. The concept of directly using *k*-mer frequency for pairwise sequence data comparison has been well explored by Nordstrom et. al. [106] to identify mutation between mutant and wild-type individuals. The *k*-mer approach is more specific than traditional hybridisation based methods of measuring the transcript abundance and it presents a greater accuracy for dinoflagellates, which consist of multi-copy gene families and pseudogenes [107].

**Figure 6:**
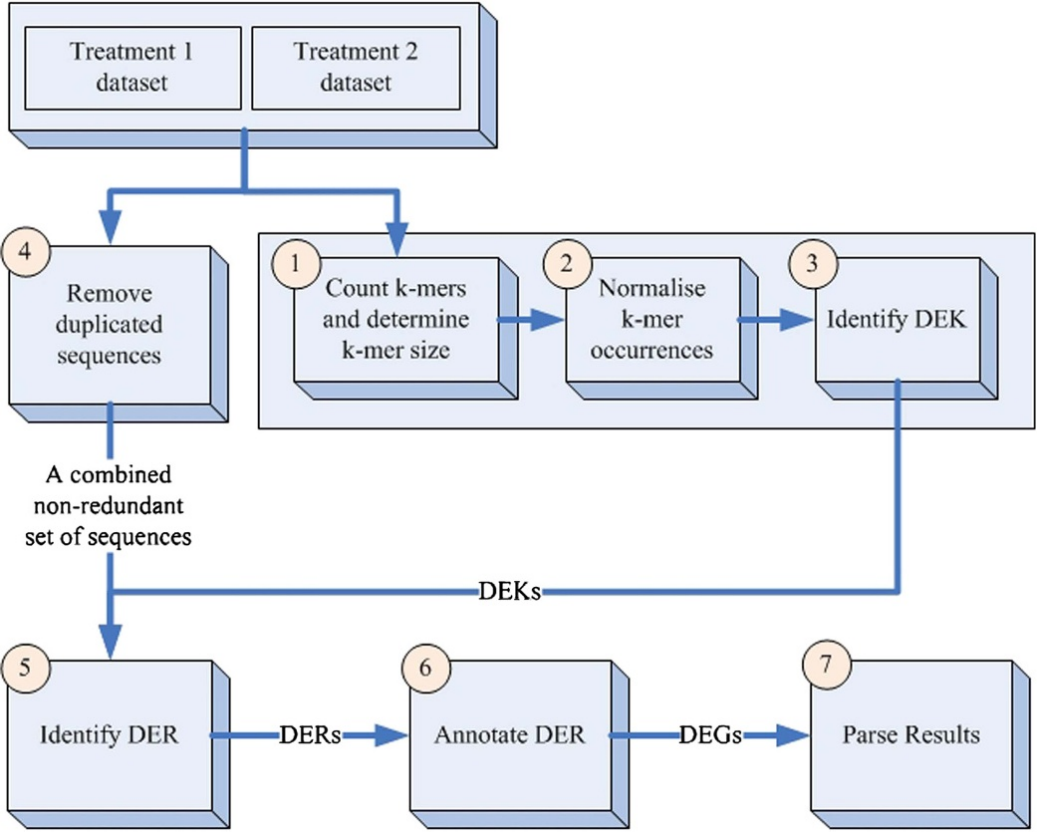
**The DiffKAP pipeline.** The schematic diagram of DiffKAP. The pipeline includes the following steps: 1- Produce a *k*-mer uniqueness plot to predict the optimum *k*-mer length; 2 - For each dataset, count the total number of each unique *k*-mer with the optimum length and normalise by dataset size; 3- Identify differentially expressed *k*-mers (DEKs) by comparing abundance between datasets; 4 - Combine the original read datasets to produce a single set of unique reads; 5 - Identify differentially expressed reads (DERs) based on their composition of DEKs; 6 - The DERs are annotated to present a redundant set of differentially expressed genes (DEGs) and 7 – Finally parse the blastx output file.

The DiffKAP pipeline analyses a pairwise dataset by identifying differentially expressed *k*-mers (DEKs) first, then obtaining differentially expressed reads (DERs) and finally annotating the DERs to gather differentially expressed genes (DEGs). It consists of seven core processing steps, as shown in Figure 6:

Step 1: DiffKAP applies Jellyfish [108] to perform *k*-mer counting. Optimal *k*-mer size is determined by adapting the strategy of Kurt et al. [109] to identify the ‘knee point’ in a *k*-mer uniqueness graph. The *k*-mer size at this point balances the specificity and sensitivity of the information content.

Step 2: The abundance of each *k*-mer is normalized by dataset size.

Step 3: DEKs are determined using the following formulas:


Where *O*_*T1*_ and *O*_*T2*_ represent the normalised *k*-mer occurrence in datasets 1 and 2 respectively, *X* represents the minimum difference of the *k*-mer occurrence and *Y* is the minimum fold change of *k*-mer occurrence between the two datasets required to call a *k*-mer differentially expressed.

Step 4: A single set of unique reads is obtained by combining all reads in the original datasets and filtering to remove duplicated reads.

Step 5: A DER is determined by a strict criterion to minimize false positives and is defined as when all constituent *k*-mers in the read are DEKs (obtained from Step 3). The DER determination is applied to all reads in the combined non-redundant set from Step 4. For DERs, the median *k*-mer abundance is calculated for each dataset and the ratio of median *k*-mer abundance (RoM) provided as a prediction of gene expression ratio. We used a 1.5-fold change for nominated DEGs by DiffKAP. This cut-off value was chosen to include DEGs of both dinoflagellate and coral origin, as often 2 or lower fold change can be observed in *Symbiodinium* transcript abundance due to thermal stress exposure [15, 21].

Step 6: All DERs are annotated by comparison to Swiss-Prot database [110] using a strict E-value cut off of ≤10^-15^ to form a set of DEGs.

Step 7: Parsing the blastx output file to be an informative tubular format.

The DiffKAP program is available from http://appliedbioinformatics.com.au/index.php/DiffKAP.

### Taxonomic composition of metatranscriptomes

In this study we used publicly available datasets, including expressed sequence tags (ESTs), genome and transcriptome sequences. To determine the taxonomic composition of each metatranscriptome, short reads from each of the analyzed samples were aligned to sequences from Genbank databases (accessed March 2012; that included bacterial, environmental, invertebrate, plant, and viral nucleotide sequences; *A. digitifera* genome; human genome; and *Symbiodinium* ESTs); then additional *Symbiodinium* ESTs (Joint Genome Institute, University of California) and the *A. millepora* transcriptome [22, 90]; the *A. hyacinthus*, *A. tenuis* and *Porites astreoides* transcriptomes (Eli Meyer, Mikhail Matz, *et al.* data: http://www.bio.utexas.edu/research/matz_lab/); and *de novo Symbiodinium* transcriptomes from this lab [33]. Database alignments were carried out using the Nucleotide-Nucleotide BLAST software application (BLASTn, version 2.2.27+), specifying a word size of 14 and E-values ≤10^-15^.

### GO enrichment analyses

GO enrichment analyses and pathway analyses were performed using the database for annotation, visualization and integrated discovery (DAVID) and identifications of enriched biological themes and KEGG (Kyoto Encyclopedia of Genes and Genomes) pathways [111, 112]. DAVID uses the Fisher’s Exact Test to ascertain statistically significant gene enrichment for a particular pathway, and significant processes were selected based on a corrected *P*-value by Benjamini [113] with a cut-off of 0.05 and even more stringent *P*-value of 0.001. To provide information about biological functions associated with the stress response in corals we used an integrated bioinformatics approach which has been provided by GO consortium [114] and DAVID bioinformatics resources [90]. We used the GO FAT database that has been developed as a part of annotation tool within the DAVID suite [115]. This database presents a subset containing more specific GO terms and reducing redundancy in big GO datasets prior enrichment analyses [116]. A number of DEGs were used for GO analyses in all three GO structures: biological process (BP), molecular function (MF) and cellular component (CC).

### Synthesis of cDNA for quantitative PCR

Reverse transcription was performed using the QuantiTect® Reverse Transcription Procedure (Qiagen, Australia). Briefly, 0.5 μg of purified total RNA was used per reaction and incubated in gDNA Wipeout Buffer at 42°C for 2 min to eliminate traces of genomic DNA, followed by reverse transcription at the same temperature for 30 min. The cDNA obtained was used as a template in the quantitative PCR (qPCR) analysis and diluted 1:10 prior to use.

### Primer design

Sequencing primers were designed using Primer Express® Software v2.0 (Applied Biosystems, USA). Reference primers used for normalization of qPCR results in the samples of *A. aspera* were adopted from previous studies [15, 18, 57] and listed in Additional file 14: Table S11. Additional primers were designed from RNA-Seq data for the selected DEGs based on DiffKAP (Additional file 15: Table S12).

For checking the specificity of primers towards coral or algal cDNA, a standard PCR amplification was carried out using aposymbiotic coral cDNA (from the coral *A. millepora* gametes) and coral-algal cDNA mix (from *A. millepora*). PCR conditions were: initial step at 94°C for 1 min; 35 cycles of 94°C 20 s, 60°C 20 s and 72°C 1.5 min; a final extension phase at 72°C for 10 min, followed by samples storage at 4°C.

### Quantitative PCR and gene expression analysis

The quantitative PCR assays were done by an Eppendorf 5075 (Applied Biosystems, USA) robot using SYBR Green PCR master mix (Applied Biosystems, UK) in 384-well plates in a 7900HT Fast Real-time PCR System (Applied Biosystems, USA). PCR conditions were: initial denaturation of 10 min at 95°C, followed by 45 cycles of 95°C for 15 s and 60°C for 1 min. At the end, a dissociation step was included: 95°C for 2 min, 60°C for 15 s and 95°C for 15 s. The final reaction volume was 10 μl and included 300 nM of primers. All reactions were carried out in two technical replicates. The expression levels of targeted genes were quantified according to geNorm directions [117]. The relative quantification method was applied for the relative abundance estimation of analysed genes using the best reference genes that showed the most stable expression patterns and specificity for *Symbiodinium* or coral. Five biological replicates were carried out for each qPCR and the mean expression level was obtained (ratio of treatment vs. control).

From the initial pool of potential reference genes (Additional file 14: Table S11), we selected the most suitable housekeeping genes (HKG) using GeNorm software (http://medgen.ugent.be/~jvdesomp/genorm/) that showed the most stable expression pattern under the experimental conditions applied in this study. The best HKGs for *Symbiodinium* used here were *Cyclophin* (*Cyc*) and *Tubulin* (*Tub*) with *M* value 0.196 and minimum of two reference genes recommended (*V*2/3 = 0.0749). For coral gene expression analyses the best HKGs were ribosomal genes *L12* (GenBank ID: EZ024706) and *L13* (GenBank ID: EZ040625) with *M* value 0.284 and again minimum of two reference genes required for accurate normalization under our experimental conditions (*V*2/3 = 0. 211). The expression of each gene was determined from the *C*_T_ (cycles threshold) value that corresponds to the number of cycles required for the PCR amplification to reach a fixed threshold in the exponential phase [118]. A specific threshold of 0.1 was used for obtaining *C*_T_ values that were transformed into quantities using maximal PCR efficiency for each gene (E = 2). The real-time dissociation curve was used to check for the presence of a unique PCR product. The results of qPCR and *k*-mer (DiffKAP method) analyses are presented on a log_2_ scale.

### Statistical analysis

Statistical analyses were performed using STATISTICA 7.0 (Statsoft Inc., Tulsa, USA). All data were tested for normality and homogeneity of variance and where assumptions were violated; the data were corrected by transformations. Non-parametric equivalents of tests were used in cases where assumptions were violated despite transformations. A Kruskal-Wallis test was used to determine the effect of time and nutrient, long-term temperature stress on *Symbiodinium* photosynthetic activity in *A. aspera*, whilst a *t*-test was used to determine the effect of short-term stress.

## Availability of supporting data

This project was submitted to NCBI BioProject with BioProject IDs: PRJNA266455 (http://www.ncbi.nlm.nih.gov/bioproject/?term=PRJNA266455); PRJNA266456 (http://www.ncbi.nlm.nih.gov/bioproject/?term=PRJNA266456); PRJNA266457 (http://www.ncbi.nlm.nih.gov/bioproject/?term=PRJNA266457); PRJNA266458 (http://www.ncbi.nlm.nih.gov/bioproject/?term=PRJNA266458) and PRJNA266459 (http://www.ncbi.nlm.nih.gov/bioproject/?term=PRJNA266459). The raw sequencing reads are deposited in NCBI SRA (Short Read Archive; http://www.ncbi.nlm.nih.gov/sra/) with the following accession numbers: SRX752503 (for C24); SRX752504 (for C72); SRX752505 (for STE); SRX752506 (for LTE) and SRX752507 (for N).

## Electronic supplementary material

Additional file 1: **Symbiont cells density and chlorophyll a concentration.** Symbiont cells density (A) and chlorophyll a concentration (B) in the coral *A. aspera* exposed to thermal and nutrient stress conditions. All data are given as the means from five independent biological replicates ± SD. Values were considered significantly different if the *P* value was <0.05 (*). (TIFF 4 MB)

Additional file 2: Table S1: The DiffKAP run summaries for STE experiment using C24 as a control after 24 h. (DOCX 29 KB)

Additional file 3: Table S2: The DiffKAP run summaries for LTE experiment using C72 as a control after 72 h. (DOCX 28 KB)

Additional file 4: Table S3: The DiffKAP run summaries for nutreint enrichment (N) experiment using C72 as a control after 72 h. (DOCX 28 KB)

Additional file 5: Table S4: Up-regulated DEGs common in all experimental conditions. Best BLASTx hits correspond to *E* value equal to and smaller than 10^-15^. (DOCX 39 KB)

Additional file 6: Table S5: Down-regulated DEGs common in all experimental conditions. Best BLASTx hits correspond to *E* value equal to and smaller than 10^-15^. (DOCX 40 KB)

Additional file 7: **The relative gene expression.** The relative expression of coral and algal genes within the coral host *A. aspera* exposed to heat (A) and nutrient stress (B) for a 3-day period using qPCR and *k*-mer (DiffKAP method) analyses. In qPCR, data normalization of the relative quantities was done using two most stable reference genes (based on the GeNorm analysis) and all data are given as the means of values obtained from five independent biological replicates. From *k*-mer analysis, the fold of change (FoC) for DEGs is calculated from the Ratio of Median (RoM) from a pool of five biological replicates with a cut-off value of 1.5-fold change (treatment vs. control). Results were presented on Log_2_ scale as Treatment versus CTRL ratio. (TIFF 6 MB)

Additional file 8: Table S6: Biological processes (BP) that have been induced by thermal (1-day and 3-day) and nutrient stress listing only the top 10 most enriched BP processes. (DOCX 49 KB)

Additional file 9: Table S7: Molecular functions (MF) enriched by thermal (1-day and 3-day) and nutrient stress listing only the top 10 most enriched MF per treatment. (DOCX 51 KB)

Additional file 10: Table S8: Cellular component (CC) enriched by thermal (1-day and 3-day) and nutrient stress listing only the top 10 most enriched CC per treatment. (DOCX 49 KB)

Additional file 11: Table S9: Taxonomic distribution of sequences (reads) from the coral holobiont after a 1-day thermal stress condition was obtained after aligning Illumina short reads to public sequence databases and the *Symbiodinium de novo* transcriptome assemblies from our group. (DOCX 41 KB)

Additional file 12: Table S10: Taxonomic distribution of sequences (reads) from the coral holobiont after exposure to the 3-day thermal and nutrient stress conditions was obtained after aligning Illumina short reads to public sequence databases and the *Symbiodinium de novo* transcriptome assemblies from our group. (DOCX 44 KB)

Additional file 13: **Supporting Information.** (DOCX 120 KB)

Additional file 14: Table S11: Primer sequences, amplicon length and gene accession number of *Symbiodinum* [57] and coral house-keeping genes [18] used in this study. (DOC 45 KB)

Additional file 15: Table S12: Primer sequences, the amplicon length and melting temperature for the primers used in the qPCR analyses. Primers were generated from RNA-Seq data and selected from DEGs based on the DiffKAP method. (DOC 40 KB)
