# Supplementary figures and images for: Early transcriptional changes in the reef-building coral Acropora aspera in response to thermal and nutrient stress
Source: BMC Genomics. 2014 Dec 2;15:1052. doi: 10.1186/1471-2164-15-1052 (PMC4301396; doi:10.1186/1471-2164-15-1052)

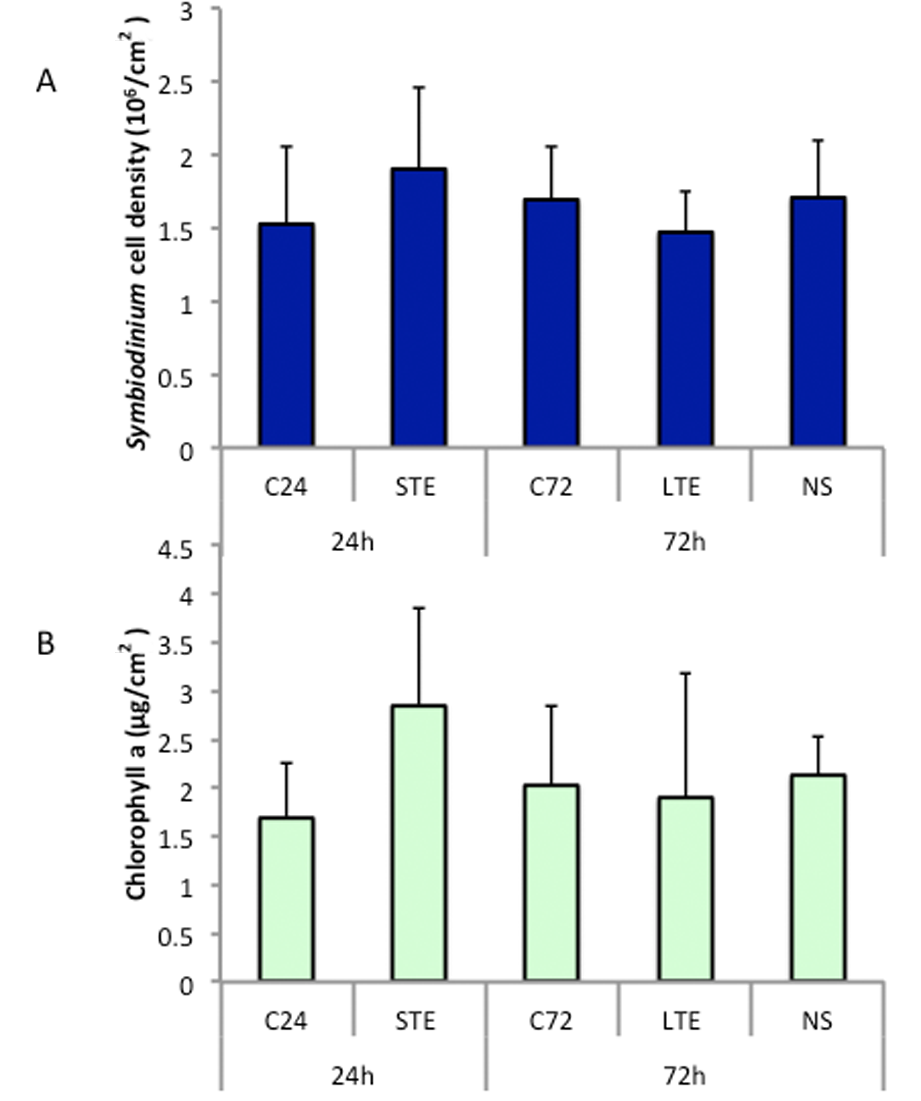

Supplement: Supplementary file 1 — Additional file 1: Symbiont cells density and chlorophyll a concentration. Symbiont cells density (A) and chlorophyll a concentration (B) in the coral A. aspera exposed to thermal and nutrient stress conditions. All data are given as the means from five independent biological replicates ± SD. Values were considered significantly different if the P value was <0.05 (*). (TIFF 4 MB) [file 12864_2014_6765_MOESM1_ESM.tiff]

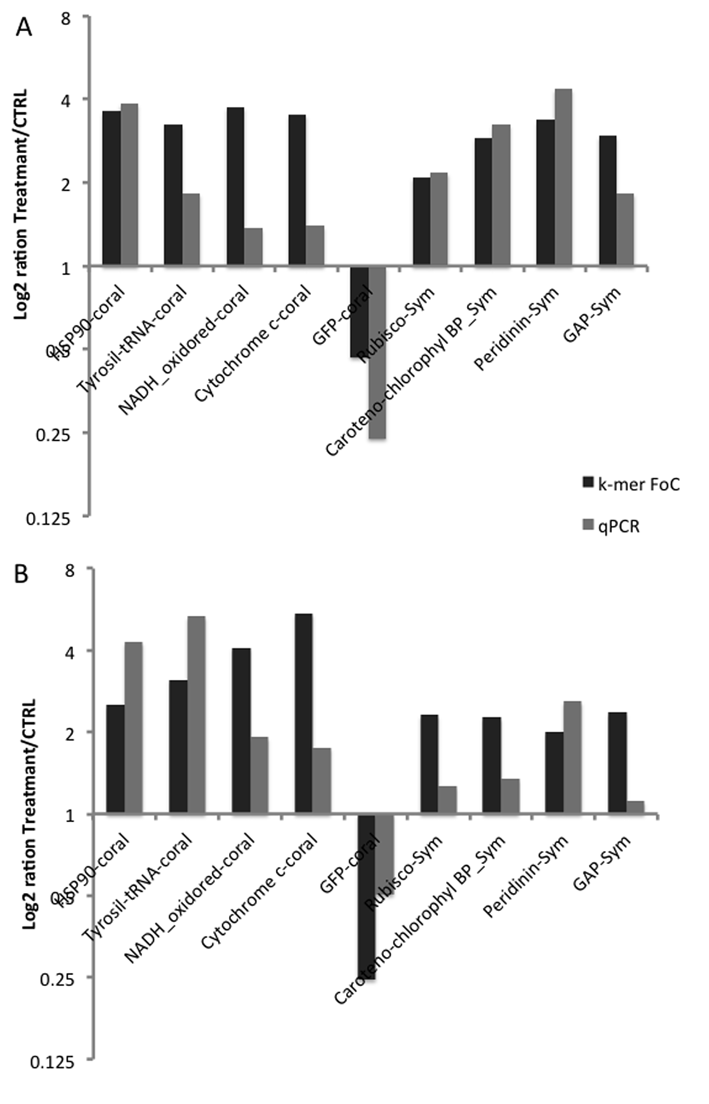

Supplement: Supplementary file 7 — Additional file 7: The relative gene expression. The relative expression of coral and algal genes within the coral host A. aspera exposed to heat (A) and nutrient stress (B) for a 3-day period using qPCR and k-mer (DiffKAP method) analyses. In qPCR, data normalization of the relative quantities was done using two most stable reference genes (based on the GeNorm analysis) and all data are given as the means of values obtained from five independent biological replicates. From k-mer analysis, the fold of change (FoC) for DEGs is calculated from the Ratio of Median (RoM) from a pool of five biological replicates with a cut-off value of 1.5-fold change (treatment vs. control). Results were presented on Log2 scale as Treatment versus CTRL ratio. (TIFF 6 MB) [file 12864_2014_6765_MOESM7_ESM.tiff]
