# Supplementary material for: Early transcriptional changes in the reef-building coral Acropora aspera in response to thermal and nutrient stress
Source: BMC Genomics. 2014 Dec 2;15:1052. doi: 10.1186/1471-2164-15-1052 (PMC4301396; doi:10.1186/1471-2164-15-1052)
Supplement: Supplementary file 2 — Additional file 2: Table S1: The DiffKAP run summaries for STE experiment using C24 as a control after 24 h. (DOCX 29 KB) [file 12864_2014_6765_MOESM2_ESM.docx]

**Table S1**

| # of read in C24 | 25549878 |
| --- | --- |
| # of read in STE | 27588956 |
| # of read in C24 & STE | 53138834 |
| # of uniq read in C24 & STE | 27792470 |
| % of uniq read in C24 & STE | 52.30% |
| Read length in C24 | 99 |
| Read length in STE | 99 |
| Kmer size used | 16 |
| Total # of kmer in C24 | 2032449864 |
| # of distinct kmer in C24 | 451665766 |
| % of distinct kmer in C24 | 22.22% |
| Total # of kmer in STE | 2316343724 |
| # of distinct kmer in STE | 195623618 |
| % of distinct kmer in STE | 8.44% |
| # of DEK | 121439780 |
| % of DEK to distinct kmer in C24 | 26.88% |
| % of DEK to distinct kmer in STE | 62.07% |
| # of DER | 1422723 |
| % of DER to uniq read | 5.11% |
| # of DER highly expressed in C24 | 585488 |
| # of DER highly expressed in STE | 658965 |
| # of annotated DER | 149027 |
| % of annotated DER | 10.47% |
| # of annotated DER highly expressed in C24 | 63601 |
| # of annotated DER highly expressed in STE | 75741 |
| Total # of DEG | 16422 |
| # of DEG with less than 10 DER | 12872 |
| % of DEG with less than 10 DER | 78.38% |
| # of DEG with 10 or more DER | 3550 |
| % of DEG with 10 or more DER | 21.61% |
