# Supplementary material for: Early transcriptional changes in the reef-building coral Acropora aspera in response to thermal and nutrient stress
Source: BMC Genomics. 2014 Dec 2;15:1052. doi: 10.1186/1471-2164-15-1052 (PMC4301396; doi:10.1186/1471-2164-15-1052)
Supplement: Supplementary file 3 — Additional file 3: Table S2: The DiffKAP run summaries for LTE experiment using C72 as a control after 72 h. (DOCX 28 KB) [file 12864_2014_6765_MOESM3_ESM.docx]

**Table S2**

| # of read in C72 | 14978045 |
| --- | --- |
| # of read in LTE | 24416729 |
| # of read in C72 & LTE | 39394774 |
| # of uniq read in C72 & LTE | 20427781 |
| % of uniq read in C72 & LTE | 51.85% |
| Read length in C72 | 99 |
| Read length in LTE | 99 |
| Kmer size used | 16 |
| Total # of kmer in C72 | 1190877840 |
| # of distinct kmer in C72 | 329422150 |
| % of distinct kmer in C72 | 27.66% |
| Total # of kmer in LTE | 2050011065 |
| # of distinct kmer in LTE | 198583522 |
| % of distinct kmer in LTE | 9.68% |
| # of DEK | 122337259 |
| % of DEK to distinct kmer in C72 | 37.13% |
| % of DEK to distinct kmer in LTE | 61.60% |
| # of DER | 781576 |
| % of DER to uniq read | 3.82% |
| # of DER highly expressed in C72 | 250597 |
| # of DER highly expressed in LTE | 425719 |
| # of annotated DER | 79484 |
| % of annotated DER | 10.16% |
| # of annotated DER highly expressed in C72 | 27652 |
| # of annotated DER highly expressed in LTE | 46705 |
| Total # of DEG | 12708 |
| # of DEG with less than 10 DER | 10833 |
| % of DEG with less than 10 DER | 85.24% |
| # of DEG with 10 or more DER | 1875 |
| % of DEG with 10 or more DER | 14.75% |
