# Supplementary material for: Early transcriptional changes in the reef-building coral Acropora aspera in response to thermal and nutrient stress
Source: BMC Genomics. 2014 Dec 2;15:1052. doi: 10.1186/1471-2164-15-1052 (PMC4301396; doi:10.1186/1471-2164-15-1052)
Supplement: Supplementary file 4 — Additional file 4: Table S3: The DiffKAP run summaries for nutreint enrichment (N) experiment using C72 as a control after 72 h. (DOCX 28 KB) [file 12864_2014_6765_MOESM4_ESM.docx]

**Table S3**

| # of read in C72 | 14978045 |
| --- | --- |
| # of read in N | 26866174 |
| # of read in C72 & N | 41844219 |
| # of uniq read in C72 & N | 22040292 |
| % of uniq read in C72 & N | 52.67% |
| Read length in C72 | 99 |
| Read length in N | 99 |
| Kmer size used | 16 |
| Total # of kmer in C72 | 1190877840 |
| # of distinct kmer in C72 | 329422150 |
| % of distinct kmer in C72 | 27.66% |
| Total # of kmer in N | 2255665321 |
| # of distinct kmer in N | 222941950 |
| % of distinct kmer in N | 9.88% |
| # of DEK | 132018037 |
| % of DEK to distinct kmer in C72 | 40.07% |
| % of DEK to distinct kmer in N | 59.21% |
| # of DER | 888826 |
| % of DER to uniq read | 4.03% |
| # of DER highly expressed in C72 | 269614 |
| # of DER highly expressed in N | 515268 |
| # of annotated DER | 84978 |
| % of annotated DER | 9.56% |
| # of annotated DER highly expressed in C72 | 31801 |
| # of annotated DER highly expressed in N | 49011 |
| Total # of DEG | 11991 |
| # of DEG with less than 10 DER | 10035 |
| % of DEG with less than 10 DER | 83.68% |
| # of DEG with 10 or more DER | 1956 |
| % of DEG with 10 or more DER | 16.31% |
