# Supplementary material for: Early transcriptional changes in the reef-building coral Acropora aspera in response to thermal and nutrient stress
Source: BMC Genomics. 2014 Dec 2;15:1052. doi: 10.1186/1471-2164-15-1052 (PMC4301396; doi:10.1186/1471-2164-15-1052)
Supplement: Supplementary file 5 — Additional file 5: Table S4: Up-regulated DEGs common in all experimental conditions. Best BLASTx hits correspond to E value equal to and smaller than 10-15. (DOCX 39 KB) [file 12864_2014_6765_MOESM5_ESM.docx]

**Table S4**

| Taxon | Accession ID | Hit description |
| --- | --- | --- |
| Fungi | P19115 | 40S ribosomal protein S14 OS=Neurospora crassa GN=rps-14 PE=3 SV=2 |
| Metazoa | P13471 | 40S ribosomal protein S14 OS=Rattus norvegicus GN=Rps14 PE=2 SV=3 |
| Viridiplantae | Q0IQF7 | 40S ribosomal protein S16 OS=Oryza sativa subsp. japonica GN=RPS16A PE=2 SV=1 |
| Metazoa | P31009 | 40S ribosomal protein S2 OS=Drosophila melanogaster GN=sop PE=1 SV=2 |
| Fungi | P33953 | 40S ribosomal protein S22 OS=Kluyveromyces marxianus GN=RPS22 PE=3 SV=3 |
| Metazoa | Q9GT45 | 40S ribosomal protein S26 OS=Anopheles gambiae GN=RpS26 PE=3 SV=2 |
| Metazoa | P48152 | 40S ribosomal protein S3 OS=Caenorhabditis elegans GN=rps-3 PE=1 SV=1 |
| Metazoa | P13195 | 5-aminolevulinate synthase, nonspecific, mitochondrial OS=Rattus norvegicus GN=Alas1 PE=2 SV=2 |
| Fungi | P53319 | 6-phosphogluconate dehydrogenase, decarboxylating 2 OS=Saccharomyces cerevisiae (strain ATCC 204508 / S288c) GN=GND2 PE=1 SV=1 |
| Metazoa | P52209 | 6-phosphogluconate dehydrogenase, decarboxylating OS=Homo sapiens GN=PGD PE=1 SV=3 |
| Metazoa | O96647 | 60S ribosomal protein L10 OS=Bombyx mandarina GN=RpL10 PE=2 SV=1 |
| Fungi | Q7RVN0 | 60S ribosomal protein L11 OS=Neurospora crassa GN=rpl-11 PE=3 SV=2 |
| Fungi | Q10157 | 60S ribosomal protein L11 OS=Schizosaccharomyces pombe (strain ATCC 38366 / 972) GN=rpl11a PE=1 SV=1 |
| Glaucocystophyceae | P93099 | 60S ribosomal protein L13a OS=Cyanophora paradoxa PE=2 SV=1 |
| Metazoa | P32100 | 60S ribosomal protein L7 OS=Drosophila melanogaster GN=RpL7 PE=2 SV=2 |
| Metazoa | Q11212 | Actin (Fragment) OS=Spodoptera littoralis PE=2 SV=1 |
| Amoebozoa | P11426 | Actin OS=Entamoeba histolytica PE=2 SV=1 |
| Metazoa | Q5BL41 | Actin-related protein 2 OS=Xenopus tropicalis GN=actr2 PE=2 SV=1 |
| Metazoa | Q7SXW6 | Actin-related protein 2-A OS=Danio rerio GN=actr2a PE=2 SV=1 |
| Metazoa | P85970 | Actin-related protein 2/3 complex subunit 2 OS=Rattus norvegicus GN=Arpc2 PE=1 SV=1 |
| Metazoa | Q6P378 | Actin, cytoplasmic 2 OS=Xenopus tropicalis GN=actg1 PE=2 SV=1 |
| Metazoa | P69005 | Actin, cytoskeletal 2B OS=Strongylocentrotus purpuratus GN=CYIIB PE=2 SV=1 |
| Metazoa | P18499 | Actin, cytoskeletal 3B OS=Strongylocentrotus purpuratus GN=CYIIIB PE=2 SV=1 |
| Firmicutes | Q9Z689 | ATP synthase subunit alpha OS=Clostridium acetobutylicum GN=atpA PE=3 SV=1 |
| Firmicutes | B1KSS6 | ATP synthase subunit alpha OS=Clostridium botulinum (strain Loch Maree / Type A3) GN=atpA PE=3 SV=1 |
| Chlamydiae | Q6MAK5 | ATP synthase subunit alpha OS=Protochlamydia amoebophila (strain UWE25) GN=atpA PE=3 SV=1 |
| Viridiplantae | Q32RL1 | ATP synthase subunit alpha, chloroplastic OS=Zygnema circumcarinatum GN=atpA PE=3 SV=1 |
| Proteobacteria | P62594 | Beta-lactamase TEM OS=Salmonella typhi GN=bla PE=3 SV=1 |
| Metazoa | P09456 | cAMP-dependent protein kinase type I-alpha regulatory subunit OS=Rattus norvegicus GN=Prkar1a PE=2 SV=2 |
| Metazoa | P00450 | Ceruloplasmin OS=Homo sapiens GN=CP PE=1 SV=1 |
| Metazoa | P82600 | Chorion peroxidase OS=Aedes aegypti GN=pxt PE=1 SV=3 |
| Metazoa | Q8SJB6 | Cytochrome b OS=Acropora tenuis GN=MT-CYB PE=3 SV=1 |
| Metazoa | O47499 | Cytochrome b OS=Metridium senile GN=MT-CYB PE=3 SV=1 |
| Viridiplantae | Q9MUV3 | Cytochrome b6 OS=Mesostigma viride GN=petB PE=3 SV=1 |
| Rhodophyta | Q6B904 | Cytochrome b6-f complex subunit 4 OS=Gracilaria tenuistipitata var. liui GN=petD PE=3 SV=1 |
| Rhodophyta | Q1XDE7 | Cytochrome b6-f complex subunit 4 OS=Porphyra yezoensis GN=petD PE=3 SV=1 |
| Cyanobacteria | Q7VDK8 | Cytochrome b6-f complex subunit 4 OS=Prochlorococcus marinus GN=petD PE=3 SV=1 |
| Viridiplantae | Q32RG3 | Cytochrome b6-f complex subunit 4 OS=Zygnema circumcarinatum GN=petD PE=3 SV=1 |
| Metazoa | Q35101 | Cytochrome c oxidase subunit 1 OS=Metridium senile GN=COI PE=3 SV=1 |
| Metazoa | Q4JQI5 | Cytochrome c oxidase subunit 1 OS=Tetraodon nigroviridis GN=mt-co1 PE=3 SV=1 |
| Viridiplantae | P68539 | Cytochrome c oxidase subunit 1 OS=Triticum aestivum GN=COX1 PE=3 SV=1 |
| Fungi | Q0H8Y4 | Cytochrome c oxidase subunit 1 OS=Ustilago maydis GN=COX1 PE=3 SV=1 |
| Metazoa | O47496 | Cytochrome c oxidase subunit 2 OS=Metridium senile GN=COII PE=3 SV=1 |
| Metazoa | O47491 | Cytochrome c oxidase subunit 3 OS=Metridium senile GN=COIII PE=3 SV=1 |
| Proteobacteria | P00582 | DNA polymerase I OS=Escherichia coli (strain K12) GN=polA PE=1 SV=1 |
| Proteobacteria | Q9F173 | DNA polymerase I OS=Salmonella typhimurium GN=polA PE=3 SV=2 |
| Metazoa | Q05052 | Dolichyl-diphosphooligosaccharide--protein glycosyltransferase 48 kDa subunit OS=Canis familiaris GN=DDOST PE=1 SV=1 |
| Metazoa | Q9ULT8 | E3 ubiquitin-protein ligase HECTD1 OS=Homo sapiens GN=HECTD1 PE=1 SV=3 |
| Proteobacteria | A0LIH6 | Elongation factor Tu OS=Syntrophobacter fumaroxidans (strain DSM 10017 / MPOB) GN=tuf1 PE=3 SV=1 |
| Metazoa | P41969 | ETS domain-containing protein Elk-1 OS=Mus musculus GN=Elk1 PE=2 SV=2 |
| Metazoa | P14315 | F-actin-capping protein subunit beta isoforms 1 and 2 OS=Gallus gallus GN=CAPZB PE=1 SV=3 |
| Metazoa | P48603 | F-actin-capping protein subunit beta OS=Drosophila melanogaster GN=cpb PE=1 SV=1 |
| Metazoa | Q9NVQ4 | Fas apoptotic inhibitory molecule 1 OS=Homo sapiens GN=FAIM PE=1 SV=1 |
| Metazoa | Q8BTM8 | Filamin-A OS=Mus musculus GN=Flna PE=1 SV=4 |
| Metazoa | P26323 | Friend leukemia integration 1 transcription factor OS=Mus musculus GN=Fli1 PE=1 SV=1 |
| Metazoa | P05065 | Fructose-bisphosphate aldolase A OS=Rattus norvegicus GN=Aldoa PE=1 SV=2 |
| Fungi | Q9HFX1 | Glyceraldehyde-3-phosphate dehydrogenase OS=Ajellomyces capsulata GN=GPD PE=2 SV=1 |
| Metazoa | Q5GIS3 | Guanine nucleotide-binding protein subunit beta OS=Pinctada fucata PE=1 SV=1 |
| Proteobacteria | P03023 | Lactose operon repressor OS=Escherichia coli (strain K12) GN=lacI PE=1 SV=3 |
| Metazoa | Q6PAB3 | Malate dehydrogenase, cytoplasmic OS=Xenopus laevis GN=mdh1 PE=2 SV=1 |
| Metazoa | Q99105 | Myosin heavy chain, embryonic smooth muscle isoform (Fragment) OS=Oryctolagus cuniculus PE=2 SV=1 |
| Metazoa | Q37556 | NADH-ubiquinone oxidoreductase chain 1 OS=Metridium senile GN=ND1 PE=3 SV=1 |
| Metazoa | O47495 | NADH-ubiquinone oxidoreductase chain 2 OS=Metridium senile GN=ND2 PE=3 SV=1 |
| Metazoa | Q35100 | NADH-ubiquinone oxidoreductase chain 3 OS=Metridium senile GN=ND3 PE=3 SV=1 |
| Metazoa | Q35099 | NADH-ubiquinone oxidoreductase chain 5 OS=Metridium senile GN=ND5 PE=3 SV=1 |
| Metazoa | Q6DJS0 | NEDD4-binding protein 1 OS=Xenopus tropicalis GN=n4bp1 PE=2 SV=1 |
| Fungi | Q01490 | Peptidyl-prolyl cis-trans isomerase B OS=Orpinomyces sp. (strain PC-2) GN=CYPB PE=1 SV=1 |
| Rhodophyta | Q9TLQ5 | Photosystem I P700 chlorophyll a apoprotein A1 OS=Cyanidium caldarium GN=psaA PE=3 SV=1 |
| Alveolata | Q9XQV3 | Photosystem I P700 chlorophyll a apoprotein A1 OS=Heterocapsa triquetra GN=psaA PE=3 SV=1 |
| Alveolata | P58383 | Photosystem I P700 chlorophyll a apoprotein A2 OS=Amphidinium carterae GN=psaB PE=3 SV=2 |
| Cyanobacteria | P09193 | Photosystem II 44 kDa reaction center protein OS=Synechocystis sp. (strain ATCC 27184 / PCC 6803 / N-1) GN=psbC PE=1 SV=3 |
| Rhodophyta | Q9TM46 | Photosystem II CP43 chlorophyll apoprotein OS=Cyanidium caldarium GN=psbC PE=3 SV=1 |
| Cryptophyta | O78426 | Photosystem II CP43 chlorophyll apoprotein OS=Guillardia theta GN=psbC PE=3 SV=2 |
| Viridiplantae | Q0P3Q1 | Photosystem II CP43 chlorophyll apoprotein OS=Ostreococcus tauri GN=psbC PE=3 SV=1 |
| Rhodophyta | Q1XDD1 | Photosystem II CP43 chlorophyll apoprotein OS=Porphyra yezoensis GN=psbC PE=3 SV=1 |
| Cyanobacteria | B0C1V6 | Photosystem II D2 protein 1 OS=Acaryochloris marina (strain MBIC 11017) GN=psbD1 PE=3 SV=1 |
| Viridiplantae | Q1ACJ7 | Photosystem II D2 protein OS=Chara vulgaris GN=psbD PE=3 SV=1 |
| Cryptophyta | O78427 | Photosystem II D2 protein OS=Guillardia theta GN=psbD PE=3 SV=1 |
| Viridiplantae | Q9TL00 | Photosystem II D2 protein OS=Nephroselmis olivacea GN=psbD PE=3 SV=1 |
| stramenopiles | A0T097 | Photosystem II D2 protein OS=Phaeodactylum tricornutum (strain CCAP 1055/1) GN=psbD PE=3 SV=1 |
| Viridiplantae | Q06SH5 | Photosystem II D2 protein OS=Stigeoclonium helveticum GN=psbD PE=3 SV=1 |
| dsDNA viruses, no RNA stage | Q6H956 | Photosystem II D2 protein OS=Synechococcus phage S-RSM2 GN=psbD PE=3 SV=1 |
| dsDNA viruses, no RNA stage | Q6H952 | Photosystem II D2 protein OS=Synechococcus phage S-WHM1 GN=psbD PE=3 SV=1 |
| Cyanobacteria | Q2JKT8 | Photosystem II D2 protein OS=Synechococcus sp. (strain JA-2-3B'a(2-13)) GN=psbD1 PE=3 SV=1 |
| Cyanobacteria | Q2JRJ5 | Photosystem II D2 protein OS=Synechococcus sp. (strain JA-3-3Ab) GN=psbD1 PE=3 SV=1 |
| Cyanobacteria | A5GQK3 | Photosystem II D2 protein OS=Synechococcus sp. (strain RCC307) GN=psbD1 PE=3 SV=1 |
| Cyanobacteria | Q116J3 | Photosystem II D2 protein OS=Trichodesmium erythraeum (strain IMS101) GN=psbD PE=3 SV=1 |
| Alveolata | Q9TM69 | Photosystem Q(B) protein OS=Alexandrium tamarense GN=psbA PE=3 SV=1 |
| Alveolata | Q7HP56 | Photosystem Q(B) protein OS=Amphidinium operculatum GN=psbA PE=3 SV=1 |
| Glaucocystophyceae | P12719 | Photosystem Q(B) protein OS=Cyanophora paradoxa GN=psbA PE=3 SV=2 |
| stramenopiles | P24726 | Photosystem Q(B) protein OS=Ectocarpus siliculosus GN=psbA PE=3 SV=1 |
| Alveolata | Q9MSC1 | Photosystem Q(B) protein OS=Heterocapsa pygmaea GN=psbA PE=3 SV=1 |
| stramenopiles | A0T0G9 | Photosystem Q(B) protein OS=Phaeodactylum tricornutum (strain CCAP 1055/1) GN=psbA PE=3 SV=1 |
| Cyanobacteria | Q7TTH6 | Photosystem Q(B) protein OS=Prochlorococcus marinus (strain MIT 9313) GN=psbA1 PE=3 SV=1 |
| Viridiplantae | P27201 | Photosystem Q(B) protein OS=Spirodela oligorhiza GN=psbA PE=3 SV=3 |
| stramenopiles | A0T0W2 | Photosystem Q(B) protein OS=Thalassiosira pseudonana GN=psbA PE=3 SV=1 |
| Viridiplantae | Q32RM3 | Photosystem Q(B) protein OS=Zygnema circumcarinatum GN=psbA PE=3 SV=1 |
| Metazoa | O35245 | Polycystin-2 OS=Mus musculus GN=Pkd2 PE=1 SV=1 |
| Metazoa | A5A3E0 | POTE ankyrin domain family member F OS=Homo sapiens GN=POTEF PE=1 SV=2 |
| Proteobacteria | Q3YVA8 | Probable GTP-binding protein EngB OS=Shigella sonnei (strain Ss046) GN=engB PE=3 SV=2 |
| Metazoa | Q98TX3 | Programmed cell death protein 4 OS=Gallus gallus GN=PDCD4 PE=2 SV=1 |
| Metazoa | Q3MHN0 | Proteasome subunit beta type-6 OS=Bos taurus GN=PSMB6 PE=1 SV=1 |
| Metazoa | Q5RDN9 | Protein yippee-like 2 OS=Pongo abelii GN=YPEL2 PE=3 SV=1 |
| Metazoa | Q2VB19 | Pumilio homolog 1 OS=Gallus gallus GN=PUM1 PE=2 SV=1 |
| Alveolata | O44006 | Pyruvate kinase OS=Eimeria tenella GN=PYK PE=2 SV=1 |
| Metazoa | Q5R7L7 | Ras-related protein Rab-5C OS=Pongo abelii GN=RAB5C PE=2 SV=1 |
| Viridiplantae | Q4LB23 | S-adenosylmethionine synthase 2 OS=Hordeum vulgare GN=SAM2 PE=2 SV=1 |
| Metazoa | Q568W0 | Selenoprotein W OS=Danio rerio GN=sepw1 PE=2 SV=3 |
| Metazoa | Q9P283 | Semaphorin-5B OS=Homo sapiens GN=SEMA5B PE=2 SV=4 |
| Metazoa | Q9D883 | Splicing factor U2AF 35 kDa subunit OS=Mus musculus GN=U2af1 PE=1 SV=4 |
| Proteobacteria | P02981 | Tetracycline resistance protein, class C OS=Escherichia coli GN=tetA PE=1 SV=1 |
| Metazoa | Q5R669 | Tribbles homolog 2 OS=Pongo abelii GN=TRIB2 PE=2 SV=1 |
| Alveolata | P14642 | Tubulin alpha chain OS=Plasmodium falciparum (isolate K1 / Thailand) PE=3 SV=1 |
| stramenopiles. | P50261 | Tubulin beta-3 chain OS=Oomycete-like sp. (strain MacKay2000) GN=TUBB3 PE=3 SV=1 |
| Proteobacteria | P03850 | Uncharacterized 10.0 kDa protein OS=Escherichia coli PE=4 SV=1 |
