# Supplementary material for: Early transcriptional changes in the reef-building coral Acropora aspera in response to thermal and nutrient stress
Source: BMC Genomics. 2014 Dec 2;15:1052. doi: 10.1186/1471-2164-15-1052 (PMC4301396; doi:10.1186/1471-2164-15-1052)
Supplement: Supplementary file 6 — Additional file 6: Table S5: Down-regulated DEGs common in all experimental conditions. Best BLASTx hits correspond to E value equal to and smaller than 10-15. (DOCX 40 KB) [file 12864_2014_6765_MOESM6_ESM.docx]

**Table S5**

| Taxon | Accession ID | Hit description |
| --- | --- | --- |
| Metazoa | [O55173](http://www.ncbi.nlm.nih.gov/protein/O55173) | 3-phosphoinositide-dependent protein kinase 1 OS=Rattus norvegicus |
| Metazoa | [Q6QAP7](http://www.ncbi.nlm.nih.gov/protein/Q6QAP7) | 40S ribosomal protein S17 OS=Sus scrofa |
| Metazoa | [Q90YQ1](http://www.ncbi.nlm.nih.gov/protein/Q90YQ1) | 40S ribosomal protein S23 OS=Ictalurus punctatus |
| Metazoa | [P62268](http://www.ncbi.nlm.nih.gov/protein/P62268) | 40S ribosomal protein S23 OS=Rattus norvegicus |
| Metazoa | [O42387](http://www.ncbi.nlm.nih.gov/protein/O42387) | 40S ribosomal protein S24 OS=Takifugu rubripes |
| Metazoa | [Q71TY3](http://www.ncbi.nlm.nih.gov/protein/Q71TY3) | 40S ribosomal protein S27 OS=Rattus norvegicus |
| Metazoa | [P24051](http://www.ncbi.nlm.nih.gov/protein/P24051) | 40S ribosomal protein S27-like OS=Rattus norvegicus |
| Metazoa | [Q90YP3](http://www.ncbi.nlm.nih.gov/protein/Q90YP3) | 40S ribosomal protein S28 OS=Ictalurus punctatus |
| Metazoa | [O61590](http://www.ncbi.nlm.nih.gov/protein/O61590) | 40S ribosomal protein S28 OS=Ostertagia ostertagi |
| Metazoa | [Q9NB51](http://www.ncbi.nlm.nih.gov/protein/Q9NB51) | 40S ribosomal protein S29 OS=Culex quinquefasciatus |
| Metazoa | [Q4PM47](http://www.ncbi.nlm.nih.gov/protein/Q4PM47) | 40S ribosomal protein S29 OS=Ixodes scapularis |
| Metazoa | [Q9N3X2](http://www.ncbi.nlm.nih.gov/protein/Q9N3X2) | 40S ribosomal protein S4 OS=Caenorhabditis elegans |
| Fungi | [P05754](http://www.ncbi.nlm.nih.gov/protein/P05754) | 40S ribosomal protein S8 OS=Saccharomyces cerevisiae (strain ATCC 204508 / S288c) |
| Viridiplantae | [P41127](http://www.ncbi.nlm.nih.gov/protein/P41127) | 60S ribosomal protein L13-1 OS=Arabidopsis thaliana |
| Metazoa | [P35427](http://www.ncbi.nlm.nih.gov/protein/P35427) | 60S ribosomal protein L13a OS=Rattus norvegicus |
| Metazoa | [O46160](http://www.ncbi.nlm.nih.gov/protein/O46160) | 60S ribosomal protein L14 OS=Lumbricus rubellus |
| Fungi | [O43004](http://www.ncbi.nlm.nih.gov/protein/O43004) | 60S ribosomal protein L16-C OS=Schizosaccharomyces pombe (strain ATCC 38366 / 972) |
| Metazoa | [Q8WQI7](http://www.ncbi.nlm.nih.gov/protein/Q8WQI7) | 60S ribosomal protein L18a OS=Spodoptera frugiperda |
| Metazoa | [Q6P5L3](http://www.ncbi.nlm.nih.gov/protein/Q6P5L3) | 60S ribosomal protein L19 OS=Danio rerio |
| Metazoa | Q27140 | 60S ribosomal protein L19 OS=Danio rerio |
| Metazoa | [Q7ZYS1](http://www.ncbi.nlm.nih.gov/protein/Q7ZYS1) | 60S ribosomal protein L19 OS=Xenopus laevis |
| Metazoa | [Q6Y263](http://www.ncbi.nlm.nih.gov/protein/Q6Y263) | 60S ribosomal protein L24 OS=Pagrus major |
| Metazoa | [Q9UNX3](http://www.ncbi.nlm.nih.gov/protein/Q9UNX3) | 60S ribosomal protein L26-like 1 OS=Homo sapiens |
| Metazoa | [Q90YU1](http://www.ncbi.nlm.nih.gov/protein/Q90YU1) | 60S ribosomal protein L27 OS=Ictalurus punctatus |
| Viridiplantae | [Q02984](http://www.ncbi.nlm.nih.gov/protein/Q02984) | 60S ribosomal protein L27 OS=Pyrobotrys stellata |
| Metazoa | [P25886](http://www.ncbi.nlm.nih.gov/protein/P25886) | 60S ribosomal protein L29 OS=Rattus norvegicus |
| Metazoa | [Q9IA76](http://www.ncbi.nlm.nih.gov/protein/Q9IA76) | 60S ribosomal protein L31 OS=Paralichthys olivaceus |
| Metazoa | [Q8WRF3](http://www.ncbi.nlm.nih.gov/protein/Q8WRF3) | 60S ribosomal protein L32 OS=Apis mellifera |
| Metazoa | [Q9NB34](http://www.ncbi.nlm.nih.gov/protein/Q9NB34) | 60S ribosomal protein L34 OS=Aedes triseriatus |
| Metazoa | [Q6DER2](http://www.ncbi.nlm.nih.gov/protein/Q6DER2) | 60S ribosomal protein L36 OS=Xenopus tropicalis |
| Metazoa | [O44125](http://www.ncbi.nlm.nih.gov/protein/O44125) | 60S ribosomal protein L37 OS=Schistosoma mansoni |
| Viridiplantae | [Q8LEM8](http://www.ncbi.nlm.nih.gov/protein/Q8LEM8) | 60S ribosomal protein L37-3 OS=Arabidopsis thaliana |
| Metazoa | [Q95V84](http://www.ncbi.nlm.nih.gov/protein/Q95V84) | 60S ribosomal protein L38 OS=Branchiostoma belcheri |
| Metazoa | [Q962S4](http://www.ncbi.nlm.nih.gov/protein/Q962S4) | 60S ribosomal protein L39 OS=Spodoptera frugiperda |
| Viridiplantae | [Q8L8W6](http://www.ncbi.nlm.nih.gov/protein/Q8L8W6) | 60S ribosomal protein L39-2 OS=Arabidopsis thaliana |
| Fungi | [Q6BLK0](http://www.ncbi.nlm.nih.gov/protein/Q6BLK0) | 60S ribosomal protein L44 OS=Debaryomyces hansenii |
| Fungi | [Q6CFS7](http://www.ncbi.nlm.nih.gov/protein/Q6CFS7) | 60S ribosomal protein L44 OS=Yarrowia lipolytica |
| Viridiplantae | [Q41630](http://www.ncbi.nlm.nih.gov/protein/Q41630) | ADP,ATP carrier protein 2, mitochondrial OS=Triticum aestivum |
| Viridiplantae | [P25083](http://www.ncbi.nlm.nih.gov/protein/P25083) | ADP,ATP carrier protein, mitochondrial OS=Solanum tuberosum GN=ANT PE=2 SV=1 |
| Metazoa | [Q8JZQ2](http://www.ncbi.nlm.nih.gov/protein/Q8JZQ2) | AFG3-like protein 2 OS=Mus musculus GN=Afg3l2 PE=1 SV=1 |
| Alveolata | [Q02766](http://www.ncbi.nlm.nih.gov/protein/Q02766) | Cytochrome c oxidase subunit 1 OS=Plasmodium falciparum GN=MT-CO1 PE=3 SV=1 |
| Metazoa | [A0PJE2](http://www.ncbi.nlm.nih.gov/protein/A0PJE2) | Dehydrogenase/reductase SDR family member 12 OS=Homo sapiens GN=DHRS12 PE=2 SV=2 |
| Metazoa | [Q5RDD3](http://www.ncbi.nlm.nih.gov/protein/Q5RDD3) | Electron transfer flavoprotein-ubiquinone oxidoreductase, mitochondrial OS=Pongo abelii GN=ETFDH PE=2 SV=1 |
| Viridiplantae | [P25698](http://www.ncbi.nlm.nih.gov/protein/P25698) | Elongation factor 1-alpha OS=Glycine max GN=TEFS1 PE=3 SV=2 |
| Fungi | [P02994](http://www.ncbi.nlm.nih.gov/protein/P02994) | Elongation factor 1-alpha OS=Saccharomyces cerevisiae (strain ATCC 204508 / S288c) GN=TEF1 PE=1 SV=1 |
| Metazoa | [P26642](http://www.ncbi.nlm.nih.gov/protein/P26642) | Elongation factor 1-gamma-A OS=Xenopus laevis GN=eef1g-a PE=1 SV=1 |
| Metazoa | [Q9EQC4](http://www.ncbi.nlm.nih.gov/protein/Q9EQC4) | Elongation of very long chain fatty acids protein 4 OS=Mus musculus GN=Elovl4 PE=2 SV=1 |
| Metazoa | [O57478](http://www.ncbi.nlm.nih.gov/protein/O57478) | Ferrochelatase, mitochondrial OS=Xenopus laevis GN=fech PE=1 SV=1 |
| Viridiplantae | [P46256](http://www.ncbi.nlm.nih.gov/protein/P46256) | Fructose-bisphosphate aldolase, cytoplasmic isozyme 1 OS=Pisum sativum PE=2 SV=1 |
| Metazoa | [Q9U6Y6](http://www.ncbi.nlm.nih.gov/protein/Q9U6Y6) | GFP-like fluorescent chromoprotein amFP486 OS=Anemonia majano PE=1 SV=1 |
| Metazoa | [O18640](http://www.ncbi.nlm.nih.gov/protein/O18640) | Guanine nucleotide-binding protein subunit beta-like protein OS=Drosophila melanogaster GN=Rack1 PE=2 SV=2 |
| Metazoa | [Q9GYZ0](http://www.ncbi.nlm.nih.gov/protein/Q9GYZ0) | Kinesin-like protein KIF15 OS=Strongylocentrotus purpuratus GN=KIF15 PE=1 SV=1 |
| Metazoa | [Q6ZQ58](http://www.ncbi.nlm.nih.gov/protein/Q6ZQ58) | La-related protein 1 OS=Mus musculus GN=Larp1 PE=1 SV=2 |
| Fungi | [P34227](http://www.ncbi.nlm.nih.gov/protein/P34227) | Mitochondrial peroxiredoxin PRX1 OS=Saccharomyces cerevisiae (strain ATCC 204508 / S288c) GN=PRX1 PE=1 SV=1 |
| Diplomonadida | [P28724](http://www.ncbi.nlm.nih.gov/protein/P28724) | NADP-specific glutamate dehydrogenase OS=Giardia intestinalis PE=2 SV=1 |
| Metazoa | [Q9VXX8](http://www.ncbi.nlm.nih.gov/protein/Q9VXX8) | Probable 60S ribosomal protein L37-A OS=Drosophila melanogaster GN=RpL37a PE=3 SV=1 |
| Metazoa | [Q9W1U6](http://www.ncbi.nlm.nih.gov/protein/Q9W1U6) | Probable 60S ribosomal protein L37-B OS=Drosophila melanogaster GN=RpL37b PE=3 SV=1 |
| Metazoa | [Q6P8H8](http://www.ncbi.nlm.nih.gov/protein/Q6P8H8) | Probable dolichyl pyrophosphate Glc1Man9GlcNAc2 alpha-1,3-glucosyltransferase OS=Mus musculus GN=Alg8 PE=2 SV=1 |
| Metazoa | [Q7ZTZ2](http://www.ncbi.nlm.nih.gov/protein/Q7ZTZ2) | Probable ribosome biogenesis protein RLP24 OS=Danio rerio GN=rsl24d1 PE=2 SV=1 |
| Metazoa | [Q4V7T8](http://www.ncbi.nlm.nih.gov/protein/Q4V7T8) | Ropporin-1-like protein OS=Xenopus laevis GN=ropn1l PE=2 SV=1 |
| Viridiplantae | [O23264](http://www.ncbi.nlm.nih.gov/protein/O23264) | Selenium-binding protein 1 OS=Arabidopsis thaliana GN=SBP1 PE=1 SV=1 |
| Metazoa | [Q63553](http://www.ncbi.nlm.nih.gov/protein/Q63553) | SNF-related serine/threonine-protein kinase OS=Rattus norvegicus GN=Snrk PE=1 SV=1 |
| Metazoa | [Q5REV9](http://www.ncbi.nlm.nih.gov/protein/Q5REV9) | Sodium-dependent phosphate transport protein 2B OS=Pongo abelii GN=SLC34A2 PE=2 SV=1 |
| Metazoa | [P35317](http://www.ncbi.nlm.nih.gov/protein/P35317) | Sodium/potassium-transporting ATPase subunit alpha OS=Hydra vulgaris PE=2 SV=1 |
| Metazoa | [P05025](http://www.ncbi.nlm.nih.gov/protein/P05025) | Sodium/potassium-transporting ATPase subunit alpha OS=Torpedo californica PE=1 SV=1 |
| Metazoa | [P25489](http://www.ncbi.nlm.nih.gov/protein/P25489) | Sodium/potassium-transporting ATPase subunit alpha-1 OS=Catostomus commersonii GN=atp1a1 PE=2 SV=1 |
| Metazoa | [Q9YH26](http://www.ncbi.nlm.nih.gov/protein/Q9YH26) | Sodium/potassium-transporting ATPase subunit alpha-1 OS=Oreochromis mossambicus GN=atp1a1 PE=2 SV=2 |
| Metazoa | [P06685](http://www.ncbi.nlm.nih.gov/protein/P06685) | Sodium/potassium-transporting ATPase subunit alpha-1 OS=Rattus norvegicus GN=Atp1a1 PE=1 SV=1 |
| Metazoa | [P06687](http://www.ncbi.nlm.nih.gov/protein/P06687) | Sodium/potassium-transporting ATPase subunit alpha-3 OS=Rattus norvegicus GN=Atp1a3 PE=1 SV=2 |
| Metazoa | [P17326](http://www.ncbi.nlm.nih.gov/protein/P17326) | Sodium/potassium-transporting ATPase subunit alpha-A OS=Artemia franciscana PE=2 SV=1 |
| Metazoa | [P28774](http://www.ncbi.nlm.nih.gov/protein/P28774) | Sodium/potassium-transporting ATPase subunit alpha-B OS=Artemia franciscana PE=2 SV=1 |
| Metazoa | [P41224](http://www.ncbi.nlm.nih.gov/protein/P41224) | Thyrotroph embryonic factor OS=Rattus norvegicus GN=Tef PE=1 SV=3 |
| Metazoa | [Q8T6A5](http://www.ncbi.nlm.nih.gov/protein/Q8T6A5) | Tubulin alpha-1 chain OS=Aplysia californica PE=2 SV=1 |
| Metazoa | [P41383](http://www.ncbi.nlm.nih.gov/protein/P41383) | Tubulin alpha-2/alpha-4 chain OS=Patella vulgata GN=TUB2 PE=2 SV=1 |
| Metazoa | [P30883](http://www.ncbi.nlm.nih.gov/protein/P30883) | Tubulin beta-4 chain OS=Xenopus laevis GN=tubb4 PE=2 SV=1 |
| Metazoa | [Q9QXA5](http://www.ncbi.nlm.nih.gov/protein/Q9QXA5) | U6 snRNA-associated Sm-like protein LSm4 OS=Mus musculus GN=Lsm4 PE=2 SV=1 |
| Metazoa | [Q7ZYP0](http://www.ncbi.nlm.nih.gov/protein/Q7ZYP0) | Ubiquitin-conjugating enzyme E2 variant 2 OS=Xenopus laevis GN=ube2v2 PE=2 SV=1 |
| Metazoa | [P63081](http://www.ncbi.nlm.nih.gov/protein/P63081) | V-type proton ATPase 16 kDa proteolipid subunit OS=Rattus norvegicus GN=Atp6v0c PE=2 SV=1 |
