# Supplementary material for: Early transcriptional changes in the reef-building coral Acropora aspera in response to thermal and nutrient stress
Source: BMC Genomics. 2014 Dec 2;15:1052. doi: 10.1186/1471-2164-15-1052 (PMC4301396; doi:10.1186/1471-2164-15-1052)
Supplement: Supplementary file 8 — Additional file 8: Table S6: Biological processes (BP) that have been induced by thermal (1-day and 3-day) and nutrient stress listing only the top 10 most enriched BP processes. (DOCX 49 KB) [file 12864_2014_6765_MOESM8_ESM.docx]

**Table S6**

| Annotation term | GO ID | No of genes | Fold Enrichment | Corrected *P*-value |
| --- | --- | --- | --- | --- |
| **1-day thermal stress** | | | | |
| Lateral inhibition | GO:0046331 | 7 | 4.85 | 0.0276 |
| Response to UV-B | GO:0010224 | 15 | 3.46 | 0.0012 |
| Response to salicylic acid stimulus | GO:0009751 | 13 | 3.15 | 0.0118 |
| Tissue regeneration | GO:0042246 | 16 | 2.68 | 0.0157 |
| Receptor cell differentiation | GO:0060113 | 17 | 2.42 | 0.0288 |
| cAMP metabolic process | GO:0046058 | 19 | 2.36 | 0.0206 |
| Mechanoreceptor differentiation | GO:0042490 | 17 | 2.36 | 0.0368 |
| Protein neddylation | GO:0045116 | 17 | 2.29 | 0.0472 |
| Protein import into nucleus, docking | GO:0000059 | 25 | 2.29 | 0.0057 |
| Determination of left/right symmetry | GO:0007368 | 32 | 2.25 | 0.0010 |
| **3-day thermal stress** | | | | |
| Histone H4 acetylation | GO:0043967 | 18 | 2.23 | 0.0340 |
| Protein-chromophore linkage | GO:0018298 | 83 | 2.16 | 2.74E-12 |
| Photosynthetic electron transport in photosystem II | GO:0009772 | 72 | 2.15 | 2.35E-10 |
| Photosynthetic electron transport chain | GO:0009767 | 94 | 2.13 | 1.29E-13 |
| Sensory perception of smell | GO:0007608 | 19 | 2.11 | 0.0451 |
| Mitotic spindle elongation | GO:0000022 | 24 | 1.98 | 0.0322 |
| Spindle elongation | GO:0051231 | 24 | 1.98 | 0.0322 |
| Cell fate determination | GO:0001709 | 39 | 1.82 | 0.0065 |
| Determination of left/right symmetry | GO:0007368 | 37 | 1.75 | 0.0211 |
| Epithelial tube morphogenesis | GO:0060562 | 55 | 1.69 | 0.0026 |
| **Nutrient stress** | | | | |
| Pyrimidine base catabolic process | GO:0006208 | 10 | 3.14 | 0.0285 |
| Mitochondrial electron transport, succinate to ubiquinone | GO:0006121 | 12 | 3.02 | 0.0119 |
| Protein-chromophore linkage | GO:0018298 | 97 | 2.47 | 7.69E-19 |
| Mitotic spindle elongation | GO:0000022 | 25 | 2.36 | 0.001047745 |
| Spindle elongation | GO:0051231 | 25 | 2.36 | 0.001047745 |
| Vesicle coating | GO:0006901 | 15 | 2.36 | 0.039833639 |
| Response to amine stimulus | GO:0014075 | 22 | 2.24 | 0.006900207 |
| Regulation of generation of precursor metabolites and energy | GO:0043467 | 26 | 2.18 | 0.003216994 |
| Chemosensory behavior | GO:0007635 | 18 | 2.12 | 0.045717727 |
| Regulation of tube size | GO:0035150 | 23 | 2.11 | 0.012014985 |
