# Supplementary material for: Early transcriptional changes in the reef-building coral Acropora aspera in response to thermal and nutrient stress
Source: BMC Genomics. 2014 Dec 2;15:1052. doi: 10.1186/1471-2164-15-1052 (PMC4301396; doi:10.1186/1471-2164-15-1052)
Supplement: Supplementary file 9 — Additional file 9: Table S7: Molecular functions (MF) enriched by thermal (1-day and 3-day) and nutrient stress listing only the top 10 most enriched MF per treatment. (DOCX 51 KB) [file 12864_2014_6765_MOESM9_ESM.docx]

**Table S7**

| Annotation term | GO ID | No of genes | Fold Enrichment | P-value |
| --- | --- | --- | --- | --- |
| **1-day thermal stress** | | | | |
| Glutamate synthase activity, NADH or NADPH as acceptor | GO:0045181 | 10 | 3.754647236 | 2.01E-04 |
| Homocysteine S-methyltransferase activity | GO:0008898 | 18 | 3.514349813 | 3.79E-07 |
| Dihydropteroate synthase activity | GO:0004156 | 10 | 3.486458148 | 4.60E-04 |
| Sulfuric ester hydrolase activity | GO:0008484 | 22 | 2.619095389 | 1.22E-05 |
| Neurotransmitter:sodium symporter activity | GO:0005328 | 31 | 2.440520704 | 8.54E-07 |
| S-methyltransferase activity | GO:0008172 | 19 | 2.37794325 | 2.79E-04 |
| Neurotransmitter transporter activity | GO:0005326 | 31 | 2.292610358 | 4.37E-06 |
| Phospholipid-translocating ATPase activity | GO:0004012 | 21 | 2.228301512 | 3.51E-04 |
| Aminophospholipid transporter activity | GO:0015247 | 21 | 2.228301512 | 3.51E-04 |
| Phospholipid transporter activity | GO:0005548 | 28 | 2.0398382 | 1.68E-04 |
| **3-day thermal stress** | | | | |
| Chlorophyll binding | GO:0016168 | 63 | 2.456239894 | 2.23E-16 |
| Oxoglutarate dehydrogenase (succinyl-transferring) activity | GO:0004591 | 21 | 2.343309325 | 2.75E-05 |
| Homocysteine S-methyltransferase activity | GO:0008898 | 18 | 2.329918986 | 1.47E-04 |
| Electron transporter, transferring electrons within the cyclic electron transport pathway of photosynthesis activity | GO:0045156 | 81 | 2.202654503 | 3.92E-16 |
| Succinate dehydrogenase (ubiquinone) activity | GO:0008177 | 26 | 2.157332394 | 1.95E-05 |
| Oxidoreductase activity, acting on the CH-CH group of donors, quinone or related compound as acceptor | GO:0016635 | 26 | 2.157332394 | 1.95E-05 |
| Electron transporter, transferring electrons within cytochrome b6/f complex of photosystem II activity | GO:0045158 | 21 | 2.059271831 | 3.99E-04 |
| ADP binding | GO:0043531 | 24 | 2.043788584 | 1.52E-04 |
| Thiamin pyrophosphate binding | GO:0030976 | 29 | 2.040086068 | 2.56E-05 |
| Calcium-transporting ATPase activity | GO:0005388 | 28 | 1.887665845 | 2.35E-04 |
| **Nutrient stress** | | | | |
| Chlorophyll binding | GO:0016168 | 75 | 2.825882418 | 4.52E-23 |
| Oxidoreductase activity, acting on the CH-CH group of donors, quinone or related compound as acceptor | GO:0016635 | 29 | 2.731686338 | 1.17E-08 |
| Succinate dehydrogenase (ubiquinone) activity | GO:0008177 | 29 | 2.731686338 | 1.17E-08 |
| Sodium:potassium-exchanging ATPase activity | GO:0005391 | 16 | 2.411419664 | 4.33E-04 |
| Electron transporter, transferring electrons within cytochrome b6/f complex of photosystem II activity | GO:0045158 | 25 | 2.242763824 | 2.70E-05 |
| Succinate dehydrogenase activity | GO:0000104 | 37 | 2.212860307 | 2.82E-07 |
| Electron transporter, transferring electrons within the cyclic electron transport pathway of photosynthesis activity | GO:0045156 | 86 | 2.077144342 | 1.26E-13 |
| Mannosidase activity | GO:0015923 | 24 | 2.055187213 | 2.48E-04 |
| Scavenger receptor activity | GO:0005044 | 44 | 2.046729653 | 3.49E-07 |
| Oxidoreductase activity, acting on the aldehyde or oxo group of donors, disulfide as acceptor | GO:0016624 | 33 | 1.802011977 | 3.10E-04 |
