# Supplementary material for: Early transcriptional changes in the reef-building coral Acropora aspera in response to thermal and nutrient stress
Source: BMC Genomics. 2014 Dec 2;15:1052. doi: 10.1186/1471-2164-15-1052 (PMC4301396; doi:10.1186/1471-2164-15-1052)
Supplement: Supplementary file 10 — Additional file 10: Table S8: Cellular component (CC) enriched by thermal (1-day and 3-day) and nutrient stress listing only the top 10 most enriched CC per treatment. (DOCX 49 KB) [file 12864_2014_6765_MOESM10_ESM.docx]

**Table S8**

| Annotation term | GO ID | No of genes | Fold Enrichment | P-value |
| --- | --- | --- | --- | --- |
| **1-day thermal stress** | | | | |
| Axonemal dynein complex | GO:0005858 | 10 | 3.104400568 | 0.001136687 |
| Axoneme part | GO:0044447 | 15 | 2.716350497 | 2.05E-04 |
| Axoneme | GO:0005930 | 55 | 2.489987955 | 2.06E-12 |
| Cilium axoneme | GO:0035085 | 35 | 2.376806685 | 1.63E-07 |
| Dynein complex | GO:0030286 | 50 | 2.336645589 | 4.78E-10 |
| Methyltransferase complex | GO:0034708 | 19 | 2.2318123 | 5.57E-04 |
| Histone methyltransferase complex | GO:0035097 | 19 | 2.2318123 | 5.57E-04 |
| Apical junction complex | GO:0043296 | 28 | 1.901445348 | 4.98E-04 |
| Occluding junction | GO:0070160 | 24 | 1.896506529 | 0.001458042 |
| Cilium part | GO:0044441 | 44 | 1.89337698 | 9.69E-06 |
| **3-day thermal stress** | | | | |
| Collagen type IV | GO:0005587 | 13 | 2.4053133 | 9.02E-04 |
| Cilium membrane | GO:0060170 | 16 | 2.36830848 | 1.96E-04 |
| Light-harvesting complex | GO:0030076 | 78 | 1.92425064 | 9.19E-12 |
| Dynein complex | GO:0030286 | 55 | 1.850241 | 1.06E-07 |
| Plasma membrane light-harvesting complex | GO:0030077 | 50 | 1.762134286 | 3.34E-06 |
| Plasma membrane-derived chromatophore | GO:0042716 | 50 | 1.762134286 | 3.34E-06 |
| Cilium axoneme | GO:0035085 | 33 | 1.684357324 | 6.48E-04 |
| Axoneme | GO:0005930 | 50 | 1.682037273 | 1.95E-05 |
| Photosystem II | GO:0009523 | 92 | 1.670892486 | 5.65E-09 |
| Cilium part | GO:0044441 | 53 | 1.669153583 | 1.41E-05 |
| **Nutrient stress** | | | | |
| Calcium- and calmodulin-dependent protein kinase complex | GO:0005954 | 10 | 2.84442477 | 0.001396503 |
| Axonemal dynein complex | GO:0005858 | 11 | 2.681886212 | 0.001354062 |
| Ribonucleoside-diphosphate reductase complex | GO:0005971 | 16 | 2.482407072 | 1.82E-04 |
| Axoneme part | GO:0044447 | 15 | 2.226071559 | 0.001611502 |
| Dynein complex | GO:0030286 | 55 | 2.085911498 | 1.33E-09 |
| Cilium axoneme | GO:0035085 | 35 | 1.958456399 | 1.43E-05 |
| Axoneme | GO:0005930 | 51 | 1.87181501 | 6.64E-07 |
| Cilium part | GO:0044441 | 53 | 1.865004282 | 4.51E-07 |
| Light-harvesting complex | GO:0030076 | 83 | 1.827772304 | 5.90E-10 |
| Photosystem I | GO:0009522 | 40 | 1.665029134 | 3.56E-04 |
