# Supplementary material for: Early transcriptional changes in the reef-building coral Acropora aspera in response to thermal and nutrient stress
Source: BMC Genomics. 2014 Dec 2;15:1052. doi: 10.1186/1471-2164-15-1052 (PMC4301396; doi:10.1186/1471-2164-15-1052)
Supplement: Supplementary file 11 — Additional file 11: Table S9: Taxonomic distribution of sequences (reads) from the coral holobiont after a 1-day thermal stress condition was obtained after aligning Illumina short reads to public sequence databases and the Symbiodinium de novo transcriptome assemblies from our group. (DOCX 41 KB) [file 12864_2014_6765_MOESM11_ESM.docx]

**Table S9**

|  | **Control** | | **STE** | |
| --- | --- | --- | --- | --- |
| **Data Source** | **# of hits** | **% of hits** | **# of hits** | **% of hits** |
| *Acropora millepora* transcriptome | 4492982 | 18.47% | 5876863 | 20.10% |
| *Acropora digitifera* genome | 8819219 | 36.26% | 9745020 | 33.34% |
| *Acropora hyacinthus* transcriptome | 3177093 | 13.06% | 4106874 | 14.05% |
| *Acropora tenuis* transcriptome | 973417 | 4.00% | 1114089 | 3.81% |
| *Porites astreoides* transcriptome | 20669 | 0.08% | 22947 | 0.08% |
| **Coral total** | **17483380** | **71.87%** | **20865793** | **71.38%** |
| *Symbiodinium* ESTs sequences (JGI) | 1614049 | 6.63% | 2292774 | 7.84% |
| *Symbiodinium* GenBank nt sequences | 133511 | 0.55% | 194537 | 0.67% |
| *Symbiodinium* *de novo*-assembled transcriptomes A2 | 26025 | 0.11% | 43892 | 0.15% |
| Symbiodinium *de novo*-assembled transcriptomes B2 | 147041 | 0.60% | 247979 | 0.85% |
| *Symbiodinium* *de novo*-assembled transcriptomes C1 | 39783 | 0.16% | 67312 | 0.23% |
| *Symbiodinium* *de novo*-assembled transcriptomes AO | 375251 | 1.54% | 603242 | 2.06% |
| ***Symbiodinium* total** | **2335660** | **9.59%** | **3449736** | **11.80%** |
| GenBank invertebrate sequences | 90306 | 0.37% | 155803 | 0.53% |
| GenBank plant, fungal, and algal sequences | 86763 | 0.36% | 178417 | 0.61% |
| GenBank bacterial sequences | 8633 | 0.04% | 99417 | 0.34% |
| GenBank environmental sampling sequences | 4167 | 0.02% | 8117 | 0.03% |
| GenBank human genome | 1861 | 0.01% | 8027 | 0.03% |
| GenBank viral sequences | 779 | 0.00% | 1987 | 0.01% |
| **Other total** | **192509** | **0.80%** | **451768** | **1.55%** |
| No Hits | 4310791 | 17.72% | 4464063 | 15.27% |
| Total # of Reads | 24322340 | 100.00% | 29231360 | 100.00% |
