# Supplementary material for: Early transcriptional changes in the reef-building coral Acropora aspera in response to thermal and nutrient stress
Source: BMC Genomics. 2014 Dec 2;15:1052. doi: 10.1186/1471-2164-15-1052 (PMC4301396; doi:10.1186/1471-2164-15-1052)
Supplement: Supplementary file 12 — Additional file 12: Table S10: Taxonomic distribution of sequences (reads) from the coral holobiont after exposure to the 3-day thermal and nutrient stress conditions was obtained after aligning Illumina short reads to public sequence databases and the Symbiodinium de novo transcriptome assemblies from our group. (DOCX 44 KB) [file 12864_2014_6765_MOESM12_ESM.docx]

**Table S10**

|  | **Control** | | **LTE** | | **N** | |
| --- | --- | --- | --- | --- | --- | --- |
| **Data Source** | **# of hits** | **% of hits** | **# of hits** | **% of hits** | **# of hits** | **% of hits** |
| *Acropora millepora* transcriptome | 2553533 | 17.78% | 4727554 | 18.82% | 4945266 | 18.41% |
| *Acropora digitifera* genome | 4708669 | 32.78% | 8319506 | 33.13% | 8960266 | 33.35% |
| *Acropora tenuis* transcriptome | 552002 | 3.84% | 1014145 | 4.04% | 1059913 | 3.95% |
| *Porites astreoides* transcriptome | 11871 | 0.08% | 22765 | 0.09% | 22364 | 0.08% |
| *Acropora hyacinthus* transcriptome | 1804162 | 12.56% | 3250910 | 12.94% | 3352901 | 12.48% |
| *Symbiodinium* ESTs sequences (JGI) | 942256 | 6.56% | 1799493 | 7.16% | 2231709 | 8.31% |
| *Symbiodinium* GenBank nt sequences | 83683 | 0.58% | 174891 | 0.70% | 160289 | 0.60% |
| *Symbiodinium* de novo-assembled transcriptomes A2 | 18397 | 0.13% | 41292 | 0.16% | 50515 | 0.19% |
| *Symbiodinium* de novo-assembled transcriptomes B2 | 114774 | 0.80% | 250560 | 1.00% | 249728 | 0.93% |
| *Symbiodinium* de novo-assembled transcriptomes C1 | 27916 | 0.19% | 63581 | 0.25% | 70988 | 0.26% |
| *Symbiodinium* de novo-assembled transcriptomes AO | 297169 | 2.07% | 621576 | 2.48% | 629262 | 2.34% |
| GenBank bacterial sequences | 7866 | 0.05% | 93216 | 0.37% | 60265 | 0.22% |
| GenBank human genome | 1107 | 0.01% | 5874 | 0.02% | 8034 | 0.03% |
| GenBank invertebrate sequences | 56797 | 0.40% | 133663 | 0.53% | 126633 | 0.47% |
| GenBank viral sequences | 524 | 0.00% | 1385 | 0.01% | 1315 | 0.00% |
| GenBank environmental sampling sequences | 1773 | 0.01% | 5345 | 0.02% | 6053 | 0.02% |
| GenBank plant, fungal, and algal sequences | 61484 | 0.43% | 158193 | 0.63% | 172048 | 0.64% |
| *No Hits* | 3121400 | 21.73% | 4430141 | 17.64% | 4758625 | 17.71% |
| Total # of Reads | 14365383 | 100.00% | 25114090 | 100.00% | 26866174 | 100.00% |
