# Supplementary material for: Early transcriptional changes in the reef-building coral Acropora aspera in response to thermal and nutrient stress
Source: BMC Genomics. 2014 Dec 2;15:1052. doi: 10.1186/1471-2164-15-1052 (PMC4301396; doi:10.1186/1471-2164-15-1052)
Supplement: Supplementary file 13 — Additional file 13: Supporting Information. (DOCX 120 KB) [file 12864_2014_6765_MOESM13_ESM.docx]

**Supporting Information**

**Rosic et al.**

**SI Materials and methods**

***Experimental design***

Sub-lethal thermal stress conditions used here are demonstrative of different warming events reported on the Great Barrier Reef (GBR) prior coral bleaching [[1-3](#_ENREF_1)], with temperatures between 31 and 32°C over a 5-day period (LTE) and between 25°C and 32°C over a 24-h period (STE). To avoid coral bleaching we applied maximum of 30ºC (that was 1-2ºC lower than during bleaching events) for 1-day and 3-day periods. Furthermore, temperatures above 30ºC led to photosynthetic dysfunction in *Symbiodinium* cultures [[4](#_ENREF_4)]. In addition, the long-thermal threshold during 1980s for the reef flat at Heron Island has been reported to be ~29.5-30ºC [[5](#_ENREF_5)]. This concentration (ammonium-enrichment: 20 μM) represents the nutrient stress condition corresponding to 10-20 times greater ammonium concentration than found in nature [[6](#_ENREF_6), [7](#_ENREF_7)]. For each replicate aquarium water temperature was measured every 2 min using StowAway TidbiT Loggers (Onset Computer Corporation, USA). The maximal temperature applied here was 30ºC, which is approximately 6-7ºC higher than mean seawater temperature during the course of the experiment.

***Symbiodinum cell density and pigment composition***

The filtered pigment extraction (0.45 µm) was used for pigment separation in a Shimadzu SCL – 10 HPLC linked to a Shimadzu SPD – M10A photodiode array detector, using the column and method described by Zapata et al. (2000) [[8](#_ENREF_8)] with solutions A (methanol: acetonitrile: ammonium acetate, 50:25:25 v:v:v) and B (methanol: acetonitrile: acetone, 20:60:20 v:v:v). Pigments concentrations were obtained using the appropriate standards and normalized to the surface area (cm^2^).

***Statistical Analysis***

For gene expression analyses, data normality and homoscedasticity were confirmed using the Kolmogorov-Smirnov test and Levene’s test, respectively. Relative expression of the analyzed genes from stressed corals was compared to the control at the same time point (coral branches not exposed to elevated temperatures or nutrient stress). Based on t*-*test, values were considered significantly different if the *P* value was <0.05 (*) or <0.01 (**). Throughout the paper, values are expressed as means ± standard deviations (s.d.).

**SI References**

1. Berkelmans R: **Time-integrated thermal bleaching thresholds of reefs and their variation on the Great Barrier Reef**. *Marine Ecology Progress Series* 2002, **229**:73-82.

2. Berkelmans R, Willis BL: **Seasonal and local spatial patterns in the upper thermal limits of corals on the inshore Central Great Barrier Reef**. *Coral Reefs* 1999, **18**(3):219-228.

3. Dove S: **Scleractinian corals with photoprotective host pigments are hypersensitive to thermal bleaching**. *Marine Ecology Progress Series* 2004, **272**:99-116.

4. Iglesias-Prieto R, Matta JL, Robins WA, Trench RK: **Photosynthetic response to elevated temperature in the symbiotic dinoflagellate *Symbiodinium microadriaticum* in culture**. *Proc Natl Acad Sci U S A* 1992, **89**(21):10302-10305.

5. Potts DC, Swart PK: **Water temperature as an indicator of environmental variability on a coral reef**. *Limnology & Oceanography* 1984, **29**(3):504-516.

6. Grover R, Maguer JF, Reynaud-Vaganay S, Ferrier-Pages C: **Uptake of ammonium by the scleractinian coral *Stylophora pistillata*: Effect of feeding, light, and ammonium concentrations**. *Limnology and Oceanography* 2002, **47**(3):782-790.

7. Koop K, Booth D, Broadbent A, Brodie J, Bucher D, Capone D, Coll J, Dennison W, Erdmann M, Harrison P *et al*: **ENCORE: The effect of nutrient enrichment on coral reefs. Synthesis of results and conclusions**. *Marine Pollution Bulletin* 2001, **42**(2):91-120.

8. Zapata M, Rodríguez F, Garrido JL: **Separation of chlorophylls and carotenoids from marine phytoplankton: A new HPLC method using a reversed phase C8 column and pyridine-containing mobile phases**. *Marine Ecology Progress Series* 2000, **195**:29-45.
