# Supplementary material for: Early transcriptional changes in the reef-building coral Acropora aspera in response to thermal and nutrient stress
Source: BMC Genomics. 2014 Dec 2;15:1052. doi: 10.1186/1471-2164-15-1052 (PMC4301396; doi:10.1186/1471-2164-15-1052)
Supplement: Supplementary file 14 — Additional file 14: Table S11: Primer sequences, amplicon length and gene accession number of Symbiodinum [57] and coral house-keeping genes [18] used in this study. (DOC 45 KB) [file 12864_2014_6765_MOESM14_ESM.doc]

**Table S11**

| **Symbol** | **Forward Primer sequence (5’-3’)** | | **Reverse Primer sequence (5’-3’)** | **GeneBank Accession Number** | **Amplicon length (bp)** | **Reference** |
| --- | --- | --- | --- | --- | --- | --- |
| *Cyc* | ATGTGCCAGGGTGGAGACTT | | CCTGTGTGCTTCAGGGTGAA | EH037450 | 101 |  |
| *Cox* | GGTCATTTCCATAATAATTTCTGGTGTT | | CAAGACCTCCAAGAAGAGAAATAGATG | EH037972 | 101 |
| *Cal* | GGCACCATCACCACCAAAG | | TCTGCGTCAACCTCGTTGATC | EH037752 | 101 |
| *Tub* | TGACGCAGCAGATGTTTGATG | | CGACATACGTCCACGGAAGAG | EH037669 | 101 |
| *Rp-S4* | CCGCACAAACTGCGTGAGT | | CGCTGCATGACGATCATCTT | EH036413 | 101 |
| *SAM* | GCCTACATTTGCCGACAGATG | | AATGGCTTGGCAACACCAAT | EH036622 | 101 |
| *L12* | | CACTGGTGGTGAAGTTGGTG | TCCAGTCTTGTGTTGCCTTG | EZ024706 | 110 |  |
| *L13* | | TTACTGGGCCGTTTAGCATC | GAGCACGGAAATGAAATGGT | EZ040625 | 184 |
| *Calmodulin* | | AGGTTGACCTGCTCGTGAGT | GCTGATGCACTGATTGGTGT | EZ030237 | 104 |
| *P0* | | GAAACGTGGGCTTTGTGTTT | TTAGTTGGAATGGCCAAAGC | EZ028666 | 188 |
| *EF1-alpha* | | CCCAAAACTGTGGCTTTTGT | TGCGTCGATAAGTGTCTTGC | DY580404 | 141 |
