# Supplementary material for: Early transcriptional changes in the reef-building coral Acropora aspera in response to thermal and nutrient stress
Source: BMC Genomics. 2014 Dec 2;15:1052. doi: 10.1186/1471-2164-15-1052 (PMC4301396; doi:10.1186/1471-2164-15-1052)
Supplement: Supplementary file 15 — Additional file 15: Table S12: Primer sequences, the amplicon length and melting temperature for the primers used in the qPCR analyses. Primers were generated from RNA-Seq data and selected from DEGs based on the DiffKAP method. (DOC 40 KB) [file 12864_2014_6765_MOESM15_ESM.doc]

**Table S12**

| **Symbol**  **(C-Coral or A-algae)** | **Forward Primer sequence (5’-3’)** | **Tm**  **(ºC)** | **Reverse Primer sequence (5’-3’)** | **Tm (ºC)** | **Amplicon length (bp)** |
| --- | --- | --- | --- | --- | --- |
| *Hsp90 (C)* | CGAGCACCCTTCGATCTCTT | 57 | GAATGATGTCCTCGCAGTTGTC | 56 | 100 |
| *GFP (C)* | GCAAGCATTCCCAGATGGAA | 56 | GCAATCTCTCTCAAGACGAATGTTC | 57 | 100 |
| *NADH (C)* | GAAGCAAATAAAGCGGCCATA | 54 | AAGACCCAAAACGATCCAAAAG | 54 | 100 |
| *Tyr (C)* | TTTGTTAAGGGCACAGAATTTCAAC | 55 | ATCCTGCCTTTTTGGCATCA | 55 | 100 |
| *Cyt_c (C)* | GGAACCATATAAGAATCAAATTCTAACGTT | 55 | AGGTTGTGAGTCCTGCTTTAACAA | 57 | 100 |
| *Rubisco (A)* | CAGACCCAGCGTGGTTACACT | 59 | TCCATCTTGCCGAAGCTCAT | 56 | 100 |
| *Peridinin (A)* | CAAGGTGCCTGCGTACATGA | 58 | CTGGTTCTTCGCAACGACATC | 57 | 100 |
| *GAP (A)* | CTGTGCGTGTGCTGCAAGA | 59 | TTGGAGGTGCCATCCACAGT | 58 | 100 |
| *Car_Chl (A)* | TCACCGCTTCCCTTGCAT | 57 | AGAGTCACCAGCTGTGGCTTAAC | 59 | 100 |
